# CRISPR–Cas ribonucleoprotein mediated homology-directed repair for efficient targeted genome editing in microalgae *Nannochloropsis oceanica* IMET1

**DOI:** 10.1186/s13068-019-1401-3

**Published:** 2019-03-25

**Authors:** Mihris Ibnu Saleem Naduthodi, Prarthana Mohanraju, Christian Südfeld, Sarah D’Adamo, Maria J. Barbosa, John van der Oost

**Affiliations:** 1 Laboratory of Microbiology, Wageningen University, Stippeneng 4, 6708 PD Wageningen, The Netherlands; 2 Bioprocess Engineering Department, Wageningen University, Droevendaalsesteeg 1, 6708 PB Wageningen, The Netherlands

**Keywords:** Microalgae, Nannochloropsis, CRISPR, Cas9, Cas12a, Ribonucleoproteins, Genome editing, Homologous recombination, Homology-directed repair

## Abstract

**Background:**

Microalgae are considered as a sustainable feedstock for the production of biofuels and other value-added compounds. In particular, *Nannochloropsis* spp. stand out from other microalgal species due to their capabilities to accumulate both triacylglycerol (TAG) and polyunsaturated fatty acids (PUFAs). However, the commercialization of microalgae-derived products is primarily hindered by the high production costs compared to less sustainable alternatives. Efficient genome editing techniques leading to effective metabolic engineering could result in strains with enhanced productivities of interesting metabolites and thereby reduce the production costs. Competent CRISPR-based genome editing techniques have been reported in several microalgal species, and only very recently in *Nannochloropsis* spp. (2017). All the reported CRISPR–Cas-based systems in *Nannochloropsis* spp. rely on plasmid-borne constitutive expression of Cas9 and a specific guide, combined with repair of double-stranded breaks (DSB) by non-homologous end joining (NHEJ) for the target gene knockout.

**Results:**

In this study, we report for the first time an alternative approach for CRISPR–Cas-mediated genome editing in *Nannochloropsis* sp.; the Cas ribonucleoproteins (RNP) and an editing template were directly delivered into microalgal cells via electroporation, making Cas expression dispensable and homology-directed repair (HDR) possible with high efficiency. Apart from widely used SpCas9, Cas12a variants from three different bacterium were used for this approach. We observed that FnCas12a from *Francisella novicida* generated HDR-based targeted mutants with highest efficiency (up to 93% mutants among transformants) while AsCas12a from *Acidaminococcus* sp. resulted in the lowest efficiency. We initially show that the native homologous recombination (HR) system in *N. oceanica* IMET1 is not efficient for easy isolation of targeted mutants by HR. Cas9/sgRNA RNP delivery greatly enhanced HR at the target site, generating around 70% of positive mutant lines.

**Conclusion:**

We show that the delivery of Cas RNP by electroporation can be an alternative approach to the presently reported plasmid-based Cas9 method for generating mutants of *N. oceanica*. The co-delivery of Cas-RNPs along with a dsDNA repair template efficiently enhanced HR at the target site, resulting in a remarkable higher percentage of positive mutant lines. Therefore, this approach can be used for efficient generation of targeted mutants in *Nannochloropsis* sp. In addition, we here report the activity of several Cas12a homologs in *N. oceanica* IMET1, identifying FnCas12a as the best performer for high efficiency targeted genome editing.

**Electronic supplementary material:**

The online version of this article (10.1186/s13068-019-1401-3) contains supplementary material, which is available to authorized users.

## Background

Microalgae have the ability to combine photosynthesis with high lipid and biomass productivities. Moreover, understanding the potential of these solar powered cell factories in food, feed and bioenergy production has elevated their interest in biotechnological research. Among other microalgal species, *Nannochloropsis* spp. has attracted considerable attention owing to its oleaginous nature. The natural ability to accumulate 60% and 12% of the cell dry weight with lipids or triacylglycerol (TAG) and omega-3 poly unsaturated fatty acid (PUFA), respectively, under defined conditions has brought these genera to the spotlight of scientific exploration [1, 2]. The consumption of omega-3 fatty acids is beneficiary for human health as they prevent heart and aging-related diseases [3], while TAG can be used as a precursor for biodiesel production [4]. Currently, the main source of omega-3 PUFA for human consumption is seafood [5]. However, the contamination of seafood due to severe ocean pollution is a major concern along with the reduction in global fish population. Furthermore, the exploitation of fossil fuels and the environmental impacts of a petroleum-based society are crucial issues to be addressed. In this context, developing *Nannochloropsis* spp. as efficient production systems can contribute to establishing a sustainable bio-economy.

In spite of the apparent advantages, the major bottleneck in employing *Nannochloropsis* spp. as an economically viable production platform for TAG and PUFA is the high cost of production [6]. One of the ways to overcome this barrier is to deploy metabolic engineering for improving the oil productivity of microalgal strains. To this end, efficient genome editing tools leading to competent metabolic engineering strategies need to be developed. Genome editing by Clustered Regularly Interspaced Short Palindromic Repeats and the CRISPR associated proteins (CRISPR–Cas) has been successfully implemented in cells of a wide range of organisms, including microalgae [7–12]. The specific DNA double-stranded breaks (DSBs) generated by CRISPR RNA (crRNA)-guided Cas nucleases is repaired by either of the two pathways: non-homologous end joining (NHEJ) or homology-directed repair (HDR) [13–15]. The NHEJ is generally associated with the introduction of insertions and/or deletions (indels) of varying lengths at the DSB site, often leading to the disruption of the reading frame of the target gene. The HDR pathway results in a precise insertion or deletion at the DSB site by homologous recombination [16–18].

Since 2017, a plasmid-driven Cas9-based approach has been implemented in *Nannochloropsis* spp. for marker-less genome editing [8, 9]. The Cas9 system has also been effectively implemented for metabolic engineering of *Nannochloropsis gaditana* to enhance the lipid production [19]. Verruto et al. combined Cas9 with Cre recombinase to recycle the limited antibiotic resistance marker along with gene stacking in *N. gaditana* [8]. Recently, non-transgenic and marker-free gene disruption was achieved in *Nannochloropsis oceanica* using an episomal CRISPR–Cas system [9].

So far, all studies on genome editing in *Nannochloropsis* spp. have relied on the plasmid-based expression of Cas9 combined with NHEJ-mediated target repair [8, 9, 19, 20]. One-step delivery using Cas-ribonucleoprotein (Cas-RNP) complex harbouring pre-assembled Cas effector protein and targeting single guide RNA (sgRNA) has been applied for genome editing in microalgae *Chlamydomonas reinhardtii* and *Phaeodactylum tricornutum* [10, 12, 21–24]. The advantages of using the Cas-RNP delivery approach include (i) the dispensability of a codon harmonized *Cas* gene, as well as promoters and terminators that are active in host of interest, (ii) circumvention of the addition of ribonucleases/ribozymes required to obtain precise processing of sgRNA transcripts to facilitate the loading of Cas9 in vivo, and (iii) reduced off-target and cytotoxic effects as Cas protein is saturated with sgRNA and is transiently active before it is degraded by endogenous proteases in cells [25–27].

Homologous recombination (HR)-based genome editing has been reported in *Nannochloropsis* spp. in 2011 [28]. However, further studies did not report any application of this technique for genome editing in *Nannochloropsis* spp. We here report the development of a Cas-RNP-mediated HDR approach for genome editing in *N. oceanica* IMET1. We enhanced the native homologous recombination system of *N. oceanica* IMET1 with targeted DSB induction by Cas9 protein to facilitate HDR and obtain knockout mutants of the nitrate reductase (*NR*) gene. CRISPR–Cas12a (previously known as Cpf1), a relatively new Cas enzyme is a dual nuclease involved in crRNA processing, target-site recognition and DSB induction [29]. As Cas9 and Cas12a proteins recognize different PAM motifs, they can be used to target distinct sites. Thus, establishing the in vivo activity of Cas12a in *Nannochloropsis* spp. expands the options of available target sites for the introduction of DSB and obtain desired mutants. In addition, this could pave way for establishing plasmid-based Cas12a systems in *Nannochloropsis* spp. for multiplexed genome editing. We also compared the efficiencies of Cas12a protein variants from *Lachnospiraceae bacterium* ND2006 (LbCas12a), *Acidaminococcus* sp. BV3L6 (AsCas12a) and *Francisella novicida* (FnCas12a) with Cas9 from *Streptococcus pyogenes* in generating HDR-based NR mutants in order to elucidate the optimal enzyme variant for generating targeted mutants using the Cas RNP-based approach in *Nannochloropsis* spp.

## Results

### Homologous recombination and antibiotic-based selection for the generation of nitrate reductase (*NR*) knockouts

**Fig. 1:**
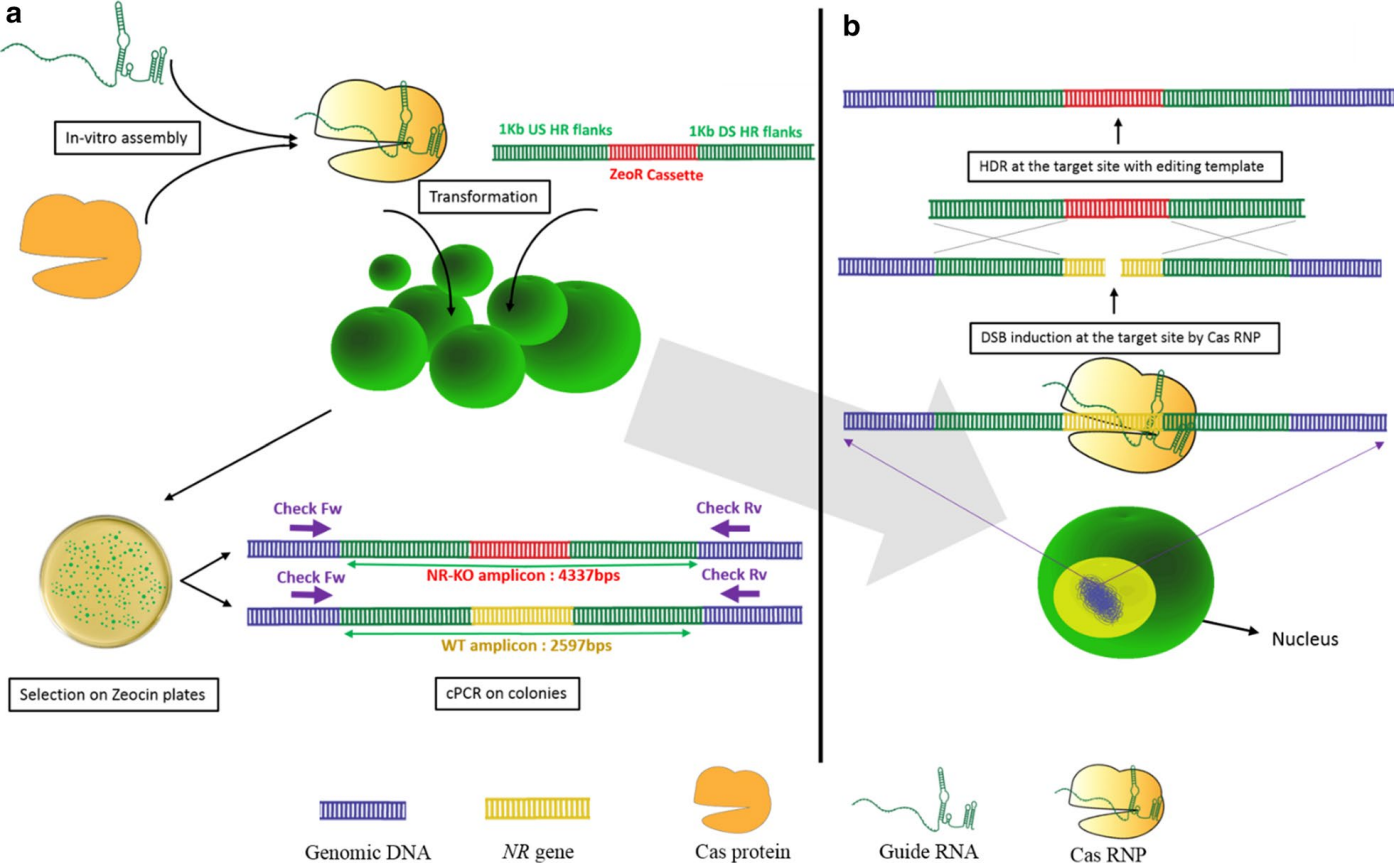
Approach; **a** the purified Cas protein is assembled with guide RNA to form the active RNP complex which is transformed along with the editing template into the competent *Nannochloropsis oceanica* IMET1 cells. The transformed cells are selected on ASW-NB plated with zeocin and ammonia and the mutants are screened by cPCR. **b** The Cas RNP introduces DSB at the target site which is repaired by HDR in presence of the editing template resulting in targeted mutants

The nitrate reductase (*NR*) gene has been used as the target for genome editing studies in *N. oceanica* as the mutants can be easily characterized through replica plating, based on their ability to grow on ammonia-containing media and their inability to grow on media containing nitrate as the sole nitrogen source [9, 20, 28]. To generate a *NR* gene knockout mutant in *N. oceanica* IMET1, we transformed cells with a vector that harbours a zeocin antibiotic resistance cassette with ~ 1 kb flanks homologous to the upstream and downstream ends of the *NR* gene locus for deletion. The 1 kb HR flanks were designed to replace 430 bp of the 519 bp long *NR* gene with a zeocin resistance cassette. After electroporation, *N. oceanica* IMET1 cells that were successfully transformed with the PCR-amplified HR editing template were selected on solid nutrient media with ammonia and 2 µg/mL zeocin. The zeocin resistant colonies were screened for targeted *NR* gene knockout via PCR. For this, we designed primers (Check FW and Check RV) binding outside the upstream and downstream of the HR flanks on the host genome (Fig. 1b). The expected PCR amplicon size of the mutant colonies generated by HDR and wild-type colonies was 4337 bp and 2591 bp, respectively.

We screened a total of 63 colonies from three independent transformation experiments with colony PCR (cPCR). For 90% colonies we observed amplicon bands corresponding to the size of the expected wild type while 10% colonies showed both wild type and mutant bands. The 10% colonies containing both the PCR amplicons are most likely the result of mutant and wild type cells at close proximity upon plating. This occurrence of 10% colonies indicates the presence of HR in *N. oceanica* IMET1. However, this Cas-independent approach is not efficient enough to facilitate routine generation of HR mutants without further optimisation.

### Cas9 RNP mediated HDR for *NR* mutant generation in *N. oceanica* IMET1

The DSB induction by endonucleases has been reported to enhance the efficiency of gene replacements by HDR in various eukaryotic cell lines [18, 30–34]. The CRISPR–Cas mediated HDR has been reported in the microalgal species *C. reinhardtii* and the diatom *Thalassiosira pseudonana* [23, 34, 35]. However, this strategy has not yet been applied in *Nannochloropsis* spp. We hypothesized that the induction of DSB using Cas9 would increase the HR efficiency in *N. oceanica.* Previous studies have shown that a plasmid-borne SpCas9/sgRNA complex can accurately generate DSBs in vivo in *N. oceanica* resulting in NHEJ mediated target gene disruption [9]. Hence, this protein was selected for exploring an RNP-based transformation approach in this species. We randomly designed 2 sgRNAs (NR-1 and NR-2), targeting the *NR* gene and transcribed them in vitro. After assessing the in vitro nuclease activity of preassembled Cas9 RNP, it was delivered along with HR editing template into *N. oceanica* IMET1 cells by electroporation (Fig. 1a). The frequency of NR mutants arising from Cas9 mediated HDR was evaluated by quantifying the percentage of expected mutants among the zeocin resistant colonies obtained after transformation.

**Fig. 2:**
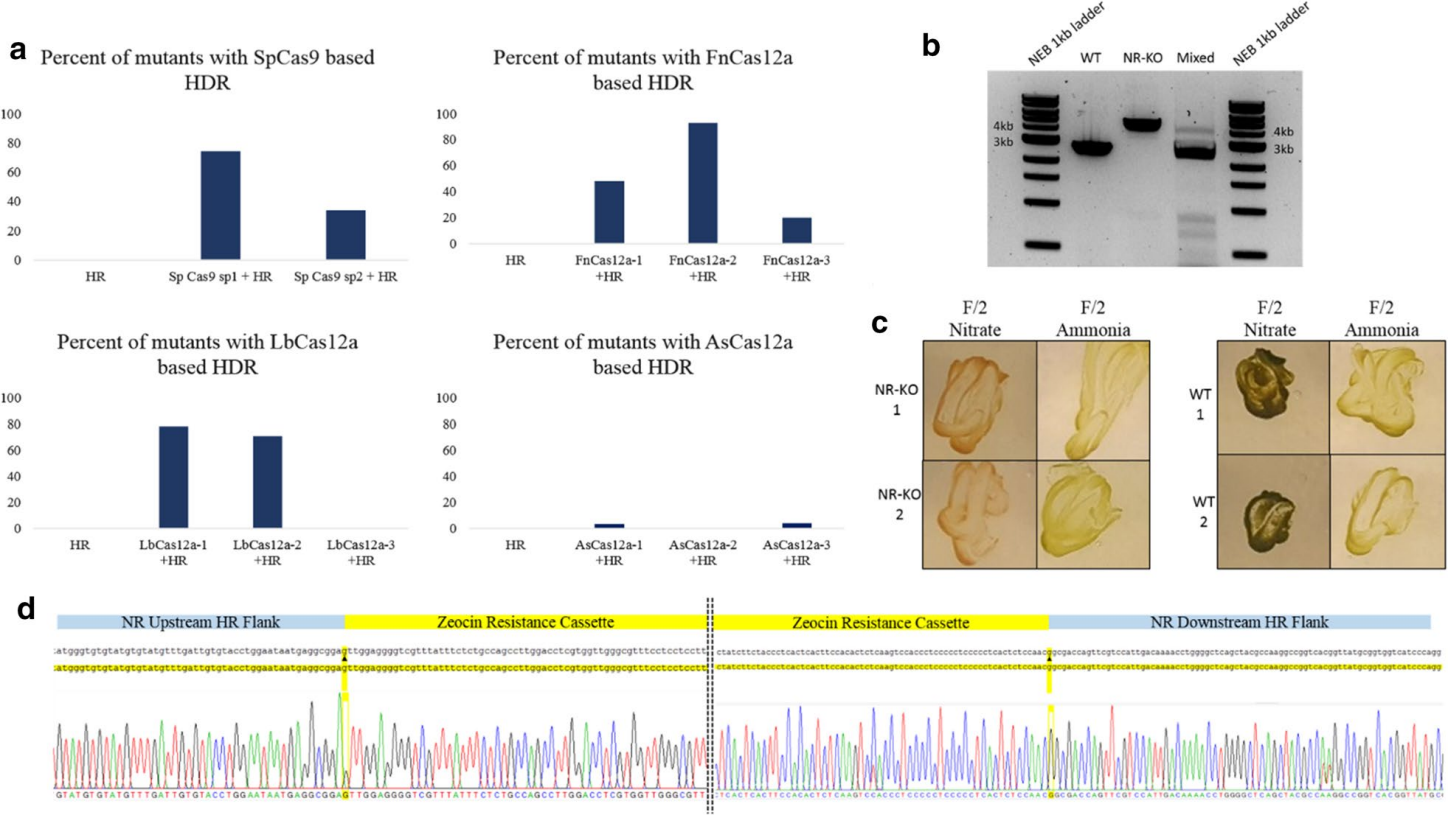
**a** Percent of mutants obtained with various RNP-based HDR. **b** Gel electrophoresis image of the WT, NR-KO and mixed colonies upon colony PCR. **c** Phenotypic characterization of NR mutants, the NR mutants were observed to bleach in media with nitrate as the sole nitrogen source while they grew similar to WT in media with ammonia as the nitrogen source (The re-streak plates for characterization included in Additional file 1: Data S13). **d** Sequencing result of a NR-KO mutant showing precise integration of the Zeocin resistance cassette into the host genome knocking out the NR gene

From three independent transformation experiments using Cas9 NR-1 and Cas9 NR-2 RNPs, a total of 32 and 35 colonies were screened, respectively, by colony PCR. The cells transfected with the Cas9/sgRNA (NR-1), we observed 12% colonies with a wild type *NR* amplicon, 15% colonies indicating the presence of both cell types, and 71% *NR* mutant colonies (Fig. 2a). As for the cells transfected with the Cas9 NR-2, 57% colonies were found to contain the wild type *NR* gene, while colonies with both amplicons (wild type and NR mutant) and NR mutant colonies reduced to 8% and 34%, respectively (Fig. 2a). HDR-based gene insertion was confirmed by sequencing 4 mutant PCR amplicons, revealing the anticipated targeted insertion of the editing template at the *NR* locus. Furthermore, the phenotypic difference between the NR mutants and the wild-type cells were demonstrated by the bleaching of NR mutant cells when streaked on F/2 agar plates with NaNO_3_ as the sole nitrogen source, but not on plates with NH_4_Cl (Fig. 2c).

### Cas12a RNP-mediated HDR for *NR* mutant generation in *N. oceanica* IMET1

To expand the genetic toolbox of *N. oceanica* IMET1 and to increase the availability of targeting sites for DSB induction, we then set out to test the efficiency of HDR using different Cas12a protein variants [*Lachnospiraceae bacterium* ND2006 (LbCas12a), *Acidaminococcus* sp. BV3L6 (AsCas12a) and *Francisella novicida* (FnCas12a)] for generating NR knockout mutants in *N. oceanica*. To this end, we designed three random crRNAs (NR-1, -2 and -3) that target the *NR* gene and were obtained as RNA molecules from Sigma Aldrich. Cas12a is guided to the target site by shorter crRNA molecules (43 nt) compared to the sgRNAs (120 nt) required for Cas9 proteins [29]. Each Cas12a protein was combined with one of the three guides, leading to a combination of 9 different Cas12a RNPs: FnCas12a NR-1, 2, 3; LbCas12a NR-1, 2, 3; AsCas12a NR-1, 2, 3. The different Cas12a RNPs along with the HR editing template were transfected into *N. oceanica* cells by electroporation. After 4 weeks, colonies were screened by cPCR.

In case of the *N. oceanica* cells transfected with FnCas12a RNPs and the HR editing template, 30 colonies per Cas12a RNP guide combination were screened. Of the 30 colonies screened, 47% of the transformants were *NR* mutants. The cells transfected with FnCas12a-NR2 and HR editing template resulted in 93% *NR* mutants, while the rest of the transformants indicated both wild type and mutant cells in them. In the case of the cells transfected with FnCas12a-3, only 20% were *NR* mutants (Fig. 2a).

For the cells transfected with LbCas12a NR1, 28 colonies were screened among which 78% were *NR* mutants while 14% were wild type. Among 28 colonies screened for the cells transfected with LbCas12a NR-2, we observed 71% mutant colonies. Upon screening 23 colonies from the cells transfected with LbCas12a NR-3, we were unable to obtain any *NR* mutant colonies (0%). However, 65% of them indicated the presence of both wild type and mutant cells and the rest of the transformants indicated wild type colonies (Fig. 2a).

We screened 27 colonies for the cells transfected with AsCas12a NR-1 and observed that 70% of them were wild type and only 3% of them were *NR* mutants. In the case of the cells transfected with AsCas12a NR-2, out of the 30 colonies screened no mutant colonies were observed (0%). Upon screening 25 colonies from the cells transfected with AsCas12a NR-3, only 4% *NR* mutants were obtained (Fig. 2a).

## Discussion

Homologous recombination and antibiotic resistance-based selection in the absence of artificially induced DSBs has been demonstrated to be highly efficient in generating genetic mutants of *N. oceanica* [28]. Our results partly agree with this observation where efficient HR is showcased in *N. oceanica* IMET1. The presence of 10% mixed colonies in our experiment indicates that the HR system is active in this species but we were not able to isolate any clean *NR* mutants by this approach. Although it might be possible to obtain isolated mutants using an HR-based approach, laborious screening of large number of colonies would be required. HR-based gene targeting has been reported in microalgal strains such as *C. reinhardtii* and *Schizochytrium* albeit it with low efficiencies (1/40–1/2000) of plasmid integration into the host genome [36–39].

As an alternative to HR for disrupting the target genes, the availability of endonucleases with tuneable sequence specificity for the introduction of DSB at specific sites and repair through NHEJ or HDR has been an important milestone in the field of genome editing [40]. The first endonucleases that were used for genome editing were zinc finger nucleases (ZFNs) and Transcription Activator Like Effector Nucleases (TALENs), but since 2013 has been dominated by the CRISPR-based nucleases for its cheap, precise and simpler approach [7, 41].

CRISPR–Cas9 based genome editing in microalgae was primarily reported in *C. reinhardtii* with a very low efficiency in generating mutants through NHEJ mediated disruption of the target gene [42]. The initial reports on *Nannochloropsis* spp. also reported similar outcome, where approximately 300 colonies were screened to obtain 2 expected mutants [20]. However, an efficient system based on Cas9 targeting and NHEJ mediated insertion of antibiotic cassette was reported in *N. gaditana* for successful disruption of up to 18 different putative transcriptional regulators [19]. In this approach, the Cas9 protein was expressed from a genome integrated plasmid that harboured a codon harmonized *Cas9* gene. The efficiency of obtaining positive knockouts in this approach varied between 6 and 78% depending on the target genes [19]. The same research team combined this Cas9 expression plasmid along with a Cre recombinase expression cassette under the control of an inducible regulator to generate markerless knockouts and recycle the antibiotic resistance for further gene knockout in the same strain [8]. Adapting the CEN/ARS6 region from *Saccharomyces cerevisiae* to develop a circular self-replicating vector active in *N. oceanica* facilitated the development of marker-free non-transgenic mutants [9]. After obtaining the expected mutants through targeted gene disruption by DSB and NHEJ repair, the mutants were grown on media without antibiotic to clear the circular plasmid from the host, thereby resulting in a non-transgenic mutant [9].

All reports to date on Cas9-based genome editing strategies in *Nannochloropsis* spp. have exploited the plasmid-based expression of Cas9 endonuclease combined with NHEJ repair systems for disruption of target genes. Our efforts to attain mutants through HR and antibiotic selection was not completely successful, but at least we obtained evidence for a functional HR system in *N. oceanica* IMET1. In the light of recently reported efficient Cas9 activity in *Nannochloropsis* spp. [8, 9, 19] and earlier reports on enhanced HR efficiency upon DSB induction [23, 33, 34, 43, 44], we applied the Cas9-based DSB induction to enhance the HR in *N. oceanica* IMET1 and thereby obtain targeted *NR* mutants.

As an alternative to the present approaches in *Nannochloropsis* spp., we used the Cas9 RNP to induce the DSB. Using a Cas RNP has some noteworthy advantages over the plasmid-based expression of Cas proteins in the host species. The off-target problem of Cas9 and strategies to overcome this issue is widely studied [45]. The transient presence of Cas RNPs in the host upon transformation reduces the risk of off-target effects compared to long-term, constitutive expression of Cas proteins from a plasmid. Moreover, the saturation of Cas protein prior to transformation with appropriate guides reduces the risk of Cas protein binding to unwanted sgRNA-like molecules leading to unspecific cleavage events. The possibility to assay the activity of RNP complex in vitro prior to tedious in vivo experiments reduces the risk of experiment failure adding to the advantages of this approach. Furthermore, the possibility to avoid the requirement of gene regulators for Cas expression and RNA processing systems for precise sgRNA production in vivo further increases the simplicity of this approach.

The co-transformation of SpCas9 RNP targeting the *NR* gene along with the HR editing template rapidly generated mutants by HDR. The observation of up to 74% mutants among the screened antibiotic resistant colonies indicates the excessive increase in HR upon using the SpCas9 RNP for DSB induction. Based on the positive results obtained with SpCas9, we decided to try to expand the genome editing toolbox of *N. oceanica* even further by assessing the efficiency of Cas12a variants in generating similar mutants. We have summarized the key differences of Cas9 and Cas12a in Table 1. As the Cas12a endonuclease introduce DSB 18–23 bp away from the PAM, the NHEJ repair might not disturb the PAM and seed sequence, thereby allowing Cas12a to cleave the same site again until HDR is successful. According to this hypothesis, Cas12a variants might exhibit even higher efficiencies than Cas9 in generating mutants through HDR.

**Table 1:** Key differences between the Cas9 and Cas12a CRISPR proteins

Feature	Cas9	Cas12a (Cpf1)
CRISPR enzyme	Class 2, Type II-B Molecular weight: ~ 164 kDa Endonuclease domains: RuvC and HNH	Class 2, Type V-A Molecular weight: ~ 158 kDa Endonuclease domains: RuvC only
PAM	3′ NGG	5′ TTTV
guideRNA	~ 120 nt, two RNA sequences crRNA and tracrRNA are synthetically fused to form the single guide RNA (sgRNA)	~ 43 nt, only crRNA is required
crRNA maturation	RNase III dependent	RNase III independent, autonomous processing
DSB cleavage site	3 bases upstream of the protospacer sequence PAM site often destroyed during genome editing	18–23 bases downstream of the PAM PAM site may be preserved after genome editing
DSB cleavage mechanism	Blunt end cuts	5-nt staggered end cut distal to the PAM

Among the three different Cas12a variants we tested (FnCas12a, LbCas12a and AsCas12a), a considerable variation in efficiencies were observed in generating mutants. Apart from the variations based on Cas12a protein, the randomly selected guide sequences used for targeting also made a difference. FnCas12a and LbCas12a were considerably active in generating mutants along with the guide sequences 1 and 2. FnCas12a marked the highest efficiency with 93% mutants among the 30 antibiotic resistant colonies screened. Irrespective of the guide sequence used for targeting, we did not observe efficient mutant generation when AsCas12a was used. Similarly, the guide sequence 3 exhibited inadequate activity in producing mutant colonies regardless of the Cas12a variant used for targeting. The diminished activity of AsCas12a in comparison to LbCas12a and FnCas12a in planta has been reported in various studies [24, 46–52]. It has been reported that the in vivo activity of Cas12a proteins were enhanced at higher temperatures (34 °C) and this effect was remarkable in AsCas12a [53]. This explains the diminished activity of AsCas12a in microalgae and plants grown at lower temperatures (< 28 °C) [53]. The sgRNA dependent variation in Cas9 cleavage has been reported in previous studies [54–57]. Correspondingly, we observe major variations in efficiency of mutant generation by the same Cas12a or Cas9 with different guide sequences. In mammalian cells, high throughput screening of sgRNAs was performed to develop a predictive model for effective sgRNA design [58]. Similar studies should be performed in microalgae to allow predicting effective sgRNAs for different target genes in silico to achieve even more efficient CRISPR-based genome editing.

The major bottleneck of our approach is the presence of an antibiotic resistance marker that allows for efficient selection of the desired mutants. This marker could be removed by applying the Cre-Lox approach as has been demonstrated into work in *N. gaditana* [8]. However, this system still leaves a minor scar (34 nt) at the target site. We have shown that HDR is very efficient in *Nannochloropsis* spp. Markerless mutant strains could thus be obtained by 2 subsequent rounds of transformation with alternating positive and negative selection. The RNP-based delivery approach could also be used for multiplexed genome editing as shown in *P. tricornutum* [12]. Co-transformation of multiple RNPs targeting different genomic loci along with multiple editing templates could facilitate HDR-based multiplexing. However, without a positive selection system based on toxicity as was demonstrated in *P. tricornutum* [12], we might require multiple antibiotic resistance markers to select mutants. Moreover, using RNPs for genome editing has also been considered to attain a non-GMO label in absence of transgenic sequences [59–61]. In this context, our approach could be developed towards achieving non-GMO knockout mutants with the help of high throughput selection tools such as FACS.

## Conclusion

Understanding the potential of developing industrially relevant *N. oceanica* has accelerated the studies on genome editing in this species. Alternative to the presently available strategies, we demonstrate the possibility of implementing Cas RNPs to drive targeted genome editing in combination with HDR in *N. oceanica*. The efficient HDR could be effectively used for precise knockout and in-frame knock-in of genes. A previous study reported the efficient generation of mutants by homologous recombination and antibiotic-based selection in this species [28]. Even though we were unable to confirm the results of this study, we witnessed the presence of an efficient HR system in the species. Co-transformation of Cas9 RNPs targeting the NR gene along with an HR editing template substantially enhanced the portion of mutants among antibiotic resistant colonies. This indicates the reinforcing effect of DSB for inducing HR at the target sites through HDR. In addition to establishing a Cas9 RNP-based transformation protocol in *N. oceanica*, we also demonstrate the activity of 3 different Cas12a variants. We observed that FnCas12a performed the best in generating mutants while AsCas12a was the weakest. LbCas12a exhibited an efficiency similar to Cas9 as observed in previous studies in *C. reinhardtii* [24]. The AsCas12a has reported to work remarkably well in mammalian cells while its activity is substantially lower in ectothermic species as their in vivo activity is diminished under lower temperature conditions (< 28 °C) [53]. Even though the mutants that we generated contain an antibiotic resistance marker, future studies will investigate selection systems for attaining marker-free, non-GMO, multiplexed mutants of *Nannochloropsis* spp. using high-throughput technology and insights generated within this research.

## Methods

### Strain and growth conditions

*Nannochloropsis oceanica* IMET1 was kindly provided by prof. Jian Xu (Qingdao Institute for Bioenergy and Bioprocess Technology, Chinese Academy of Sciences). Microalgal cultures were grown in artificial sea water (ASW) media composed of 24.50 g/L sodium chloride, 3.20 g/L sodium sulphate, 0.80 g/L calcium chloride di-hydrate, 0.85 g/L potassium sulphate and 9.80 g/L magnesium chloride hexahydrate. The ASW media was further enriched with commercial “nutribloom plus” (ASW-NB) obtained from Necton (Olháo, Portugal) or F/2 nutrients (ASW-F/2). When culturing with CO_2_ supplementation (5% CO_2_), the media was provided with 3 g/L Sodium bicarbonate and while culturing without CO_2_ 4.77 g/L HEPES was added to ASW and pH was set to 8.0 prior to autoclaving and nutrient supplementation. Ammonium chloride with a final concentration of 12 mM was used in the media for *NR* mutants.

### Plasmid construction

The homology repair template was designed to drive the removal of the nitrate reductase activity and in turn confer antibiotic resistance against Zeocin upon engineered cells. It carried the bleomycin resistance gene from *Streptoalloteichus hindustanus* (shble, GenBank accession number A31898.1) under the control of an endogenous promoter and terminator. The Violaxanthin Chlorophyll a Binding Protein Precursor (VCP) promoter region including the translation initiation ATG codon and the first intron of the VCP gene was amplified from genomic DNA using Q5 DNA polymerase (NEB) with the primers VCP FW and VCP RV. This promoter exhibited strong constitutive expression in transcriptomic studies [62] and a similar construct has been reported to drive *shble* expression in *N. oceanica* [63]. The *shble* gene was amplified from the plasmid pPtPuc3 (addgene #62863) which was a gift from Hamilton Smith. The gene was amplified with primer set BLE FW and BLE RV. The 3′UTR and transcriptional terminator of the alpha tubulin gene were previously used in *N. oceanica* IMET1 [20]. We amplified them with the primer set A-TUB FW and A-TUB RV. We chose to add a second transcriptional terminator in reverse orientation, which was amplified from the 3′-region of the *Clp* protease proteolytic subunit gene with primers CLP FW and CLP RV. The fragments were assembled using the Gibson assembly technique (NEBuilder^®^ HiFi DNA Assembly Master Mix) with 25 nucleotides overlap. The construct was introduced into the MCS of the cloning vector pUC19 (GenBank accession number M77789.2), which was linearized via PCR with primer set pUC FW and pUC RV. The correct assembly of these fragments in the construct pNIM14 was verified via PCR and sequencing.

In a second cloning step, ~ 1 kb homologous flanks for the knockout of the *NR* gene were added to both sides of the antibiotic resistance cassette. The upstream homologous flank was amplified from host genomic DNA with the primer set NR US Fw and NR US Rv and the downstream flank with primers NR DS Fw and NR DS Rv. The antibiotic resistance cassette was amplified by PCR with primer sets ZeoR Fw and ZeoR Rv harbouring overlaps to the US and DS HR flanks, respectively. The pUC19 plasmid was linearized with primers pUC19 Fw and pUC19 Rv harbouring the overlaps to align with the HR flanks and antibiotic resistance cassette by Gibson assembly technique. After assembling the PCR products by NEBuilder^®^ HiFi DNA Assembly Master Mix, the circular product was transformed into Dh5 alpha *E. coli* and the alignment was validated from single colonies by PCR and sequencing. The Genbank file of the vector developed is included in Additional file 1: Data S15. Once the construct was sequence-verified, the linear DNA for the transformation of microalgal cells was PCR amplified from the construct with primers NR US Fw and NR DS Rv.

### Transformation of *N. oceanica*

For transformation we followed the protocol developed by Vieler et al. The linearized vector, carrier DNA and RNPs were added into this 0.2 mL of 5 × 10^8^ cells/mL concentrated culture and electroporation was performed using a 2 mm cuvette and following pulse settings: 2000 V, 600 Ohm resistance and 50 µF capacitance. After electroporation, the cells were transferred to 5 mL ASW in 15 mL Falcon tubes supplemented with nutribloom and ammonium chloride (12 mM) and left under continuous light of 50 µmol m^−2^ s^−1^ without mixing for recovery (2 days).

The linear PCR product for transformation into *N. oceanica* IMET1 was amplified from the circular plasmids obtained from *E. coli*. Primers NR US FW and NR DS RV were used for amplifying the linear vector. 3 µg of linear vector DNA and 30 µg of Salmon Sperm DNA were used for transformation. For transformation with RNP complex, we pre-assembled purified Cas protein with appropriate guide sequences in an equimolar ratio (10 µM) and incubated at 37 °C for 10 min to form the Cas RNPs. 30 µL of 10 µM RNPs were transformed along with 3 µg editing template and 30 µg carrier DNA (Salmon sperm DNA).

The transformants were plated on ASW-NB (1% Agar), 1.2 mM Ammonium chloride and 2 µg/mL zeocin. The plates were incubated under continuous light of 50 µmol m^−2^ s^−1^ for 3–4 weeks until colonies appeared.

### sgRNA production

The sgRNA for targeting the *NR* gene with SpCas9 was produced by in vitro transcription of the corresponding DNA template with HiScribe T7 RNA synthesis from NEB. Two targeting spacers (sp1: 5′-GCCGGCGCAGACAAGAGTGA-3′ & sp2: 5′-AACCTCCTTGCGGCGATTGC-3′) were selected from the *NR* gene based on the PAM sequence 5′-NGG-3′. Following forwards primers were designed for adding the spacer sequence onto the sgRNA loop by PCR:Sp1 sgRNA FW and Sp2 sgRNA FW with common reverse primer sgRNA RV. After obtaining the PCR fragment with spacer sequence and sgRNA loop, a subsequent PCR was performed to attach the T7 promoter sequence 5′-TAATACGACTCACTATAGG-3′ to the 5′ end of the fragment. Following forward primers were used for adding the T7 promoter; T7 sp1 Fw and T7 sp2 Fw with the reverse primer sgRNA RV. The same reverse primer used in the previous PCR was used for above PCR reactions to obtain the final DNA fragment for in vitro transcription. The final DNA template sequence used for in vitro transcription is provided in Additional file 2: Table S1.

The in vitro transcription was performed according to the protocol provided by the supplier. The sgRNA was gel purified according to the protocol developed by Anders and Jinek [64] and the activity was confirmed through the following in vitro assay. The sequences of all guide RNAs and crRNAs are included in Additional file 2: Table S1.

### Cas proteins expression and purification

*Escherichia coli* codon optimized *spcas9*, *E. coli* codon harmonized *fncas12a*, and human codon optimized *ascas12a* and *lbcas12a* genes were cloned into a bacterial expression vector [6-His-TEV-Cas12a, a pET-based vector that was a gift from Scott Gradia (Addgene plasmid # 29653)]. One litre of LB growth media with 100 μg/mL ampicillin was inoculated with 10 mL overnight culture Rosetta (DE3) (EMD Millipore) cells containing the Cas12a expression construct. Growth media plus inoculant was grown at 37 °C until the cell density reached 0.5 OD_600_, then the temperature was decreased to 20 °C. Growth was continued until OD_600_ reached 0.6 when a final concentration of 500 μM IPTG was added to induce Cas12a expression. The culture was induced for 14–18 h before harvesting cells and freezing them at − 20 °C until purification.

Cell paste was suspended in 20 mL of Lysis Buffer (50 mM NaH_2_PO_4_ pH 8, 500 mM NaCl, 1 mM 2-Mercaptoethanol, 10 mM imidazole) supplemented with protease inhibitors (Roche complete, EDTA-free) and lysozyme. Once homogenized, cells were lysed by sonication (Bandelin Sonoplus) and then centrifuged at 16,000×*g* for 1 h at 4 °C to clear the lysate. The lysate was filtered through 0.22 micron filters (Mdi membrane technologies) and applied to a nickel column (Histrap HP, GE lifesciences), washed and then eluted with 250 mM imidazole. Fractions containing protein of the expected size were pooled and dialyzed overnight into the dialysis buffer (250 mM KCl, 20 mM HEPES/KOH, 1 mM DTT). After dialysis, sample was diluted 1:1 in 10 mM HEPES/KOH pH 8.0, and loaded on a heparin FF column pre-equilibrated in IEX-A buffer (150 mM KCl, 20 mM HEPES/KOH pH 8). Column was washed with IEX-A and then eluted with a gradient of IEX-C (2 M KCl, 20 mM HEPES/KOH pH 8.0). The sample was concentrated to 700 μL prior to loading on a gel filtration column (HiLoad 16/600 Superdex 200) via FPLC (AKTA Pure). Fractions from gel filtration were analyzed by SDS-PAGE; fractions containing the Cas12a1 were pooled and concentrated to 200 μL (50 mM Tris–HCl pH 8.0, 2 mM DTT, 5% glycerol, 500 mM NaCl) and either used directly for biochemical and transfection assays, or frozen at − 80 °C for storage.

### In vitro assays of RNP complex

To obtain the RNP complex, equimolar amount (1 µM) of Cas protein and sgRNA were incubated in a sterile 1.5 mL eppendorf tube at 37 °C for 15 min along with 3 µL 10X Cas buffer (NEBuffer 3.1) and milliQ water to a final volume of 30 µL. The 10X Cas buffer is composed of 1 M Nacl, 500 mM Tris–HCl, 100 mM MgCl_2_ and 1 mg/mL BSA at pH 7.9. After 15 min incubation, 100 ng of the template DNA was added into the reaction mixture and incubated again for 1 h. The target DNA was amplified from the host genomic DNA with Q5 (NEB) PCR by using primers NR Fw: 5′-GTGGTGCGTAGTCGGAATGG-3′ and NR Rv: 5′-GTCGGCCAATCCAGTTCGTGTC-3′. The template DNA was 1207 bp long and the digestion with Cas9 could result in fragments of size 183 and 1022 bp with sp1 and 200 and 1005 bp with sp2. Similarly, the cleavage with Cas12a spacer 1 generates fragments of size 314 and 898 bp, spacer 2 can produce fragments of size 443 and 774 bp while spacer 3 produces fragments of size 458 and 750 bp.

The activity of the RNP complex for cleaving the target DNA was assayed at 37 °C and 25 °C to assess the activity of RNP at the optimum temperature of Cas protein and temperature for in vivo experiment, respectively. The result of in vitro cleavage assay can be found in Additional file 1: S1.

### Colony PCR on transformants

Colony PCR was performed with the Phire Plant Direct PCR Master Mix (Thermo). Single colonies were taken with a 10 µL inoculation loops and re-streaked to a fresh plate and incubated at same conditions. Also, another portion of the same single colony was resuspended in 20 µL dilution buffer (obtained with the Phire Plant Direct PCR Master Mix kit) in an 8-strip PCR tubes. After incubating the sample for 15 min at room temperature, the sample was centrifuged for 30 s. 2 µL of the supernatant from the dilution buffer-colony mixture was used as template for the Phire Plant Direct PCR. Following primer set was used for screening the antibiotic-resistant colonies for mutants: Check FW: GTGCTTGATGCGGACGACAG, Check RV: AAAGCGCACGACGCAATGG.

## Additional files

**Additional file 1.** Supplementary file.

**Additional file 2.** Supplementary table.

## Abbreviations

CRISPR: Clustered Regularly Interspaced Short Palindromic Repeat
sgRNA: single-guide RNA
DSB: double stranded break
bp: base pairs
NR: nitrate reductase
