# Supplementary material for: CRISPR–Cas ribonucleoprotein mediated homology-directed repair for efficient targeted genome editing in microalgae Nannochloropsis oceanica IMET1
Source: Biotechnol Biofuels. 2019 Mar 25;12:66. doi: 10.1186/s13068-019-1401-3 (PMC6432748; doi:10.1186/s13068-019-1401-3)
Supplement: Supplementary file 1 — Additional file 1. Supplementary file. [file 13068_2019_1401_MOESM1_ESM.docx]

# Supplementary data

**NEB 100 bp ladder**


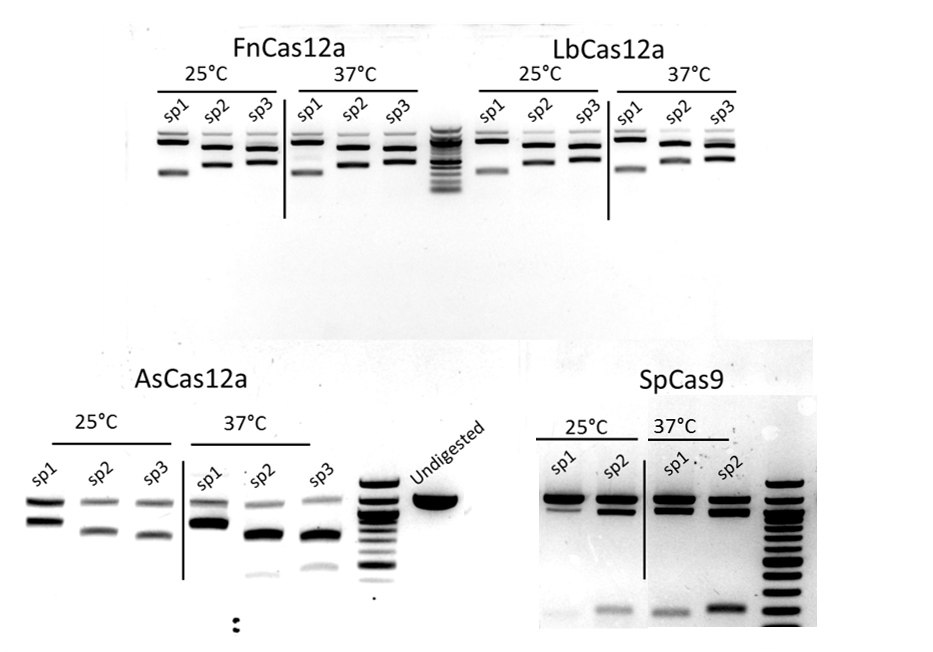


S1. In-vitro assays of various RNP complexes used in the study. The assay was performed at both 25˚C and 37˚C. The Cas12a variants are predicted to cleave the target fragment (1207 bps) into 2 smaller fragments of respective sizes based on the guide sequence; sp1: 314 bps and 893bps, sp2: 443 bps and 774 bps, sp3: 458 bps and 750bps. The Cas9 RNP with sp1 upon cleavage produced fragments of size 183 bps and 1022 bps while sp2 produced bands of size 200 bps and 1005 bps.

**NEB 100 bp ladder**

**NEB 100 bp ladder**

**NEB 100 bp ladder**

**2kb**

**3kb**


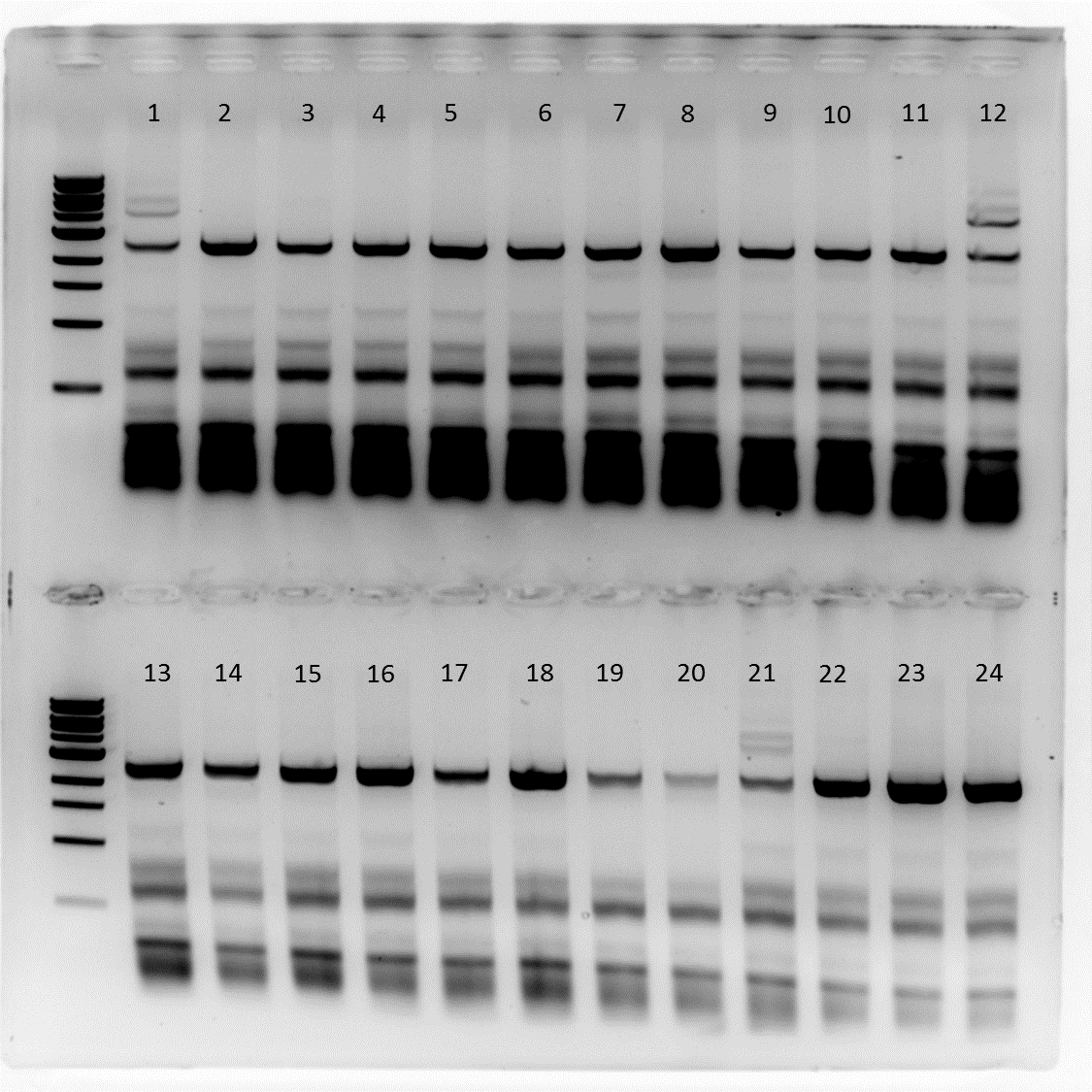


**NEB 1kb ladder**

**NEB 1kb ladder**

**4kb**

**4kb**

**3kb**

**2kb**

**2kb**

**3kb**

**4kb**

**3kb**


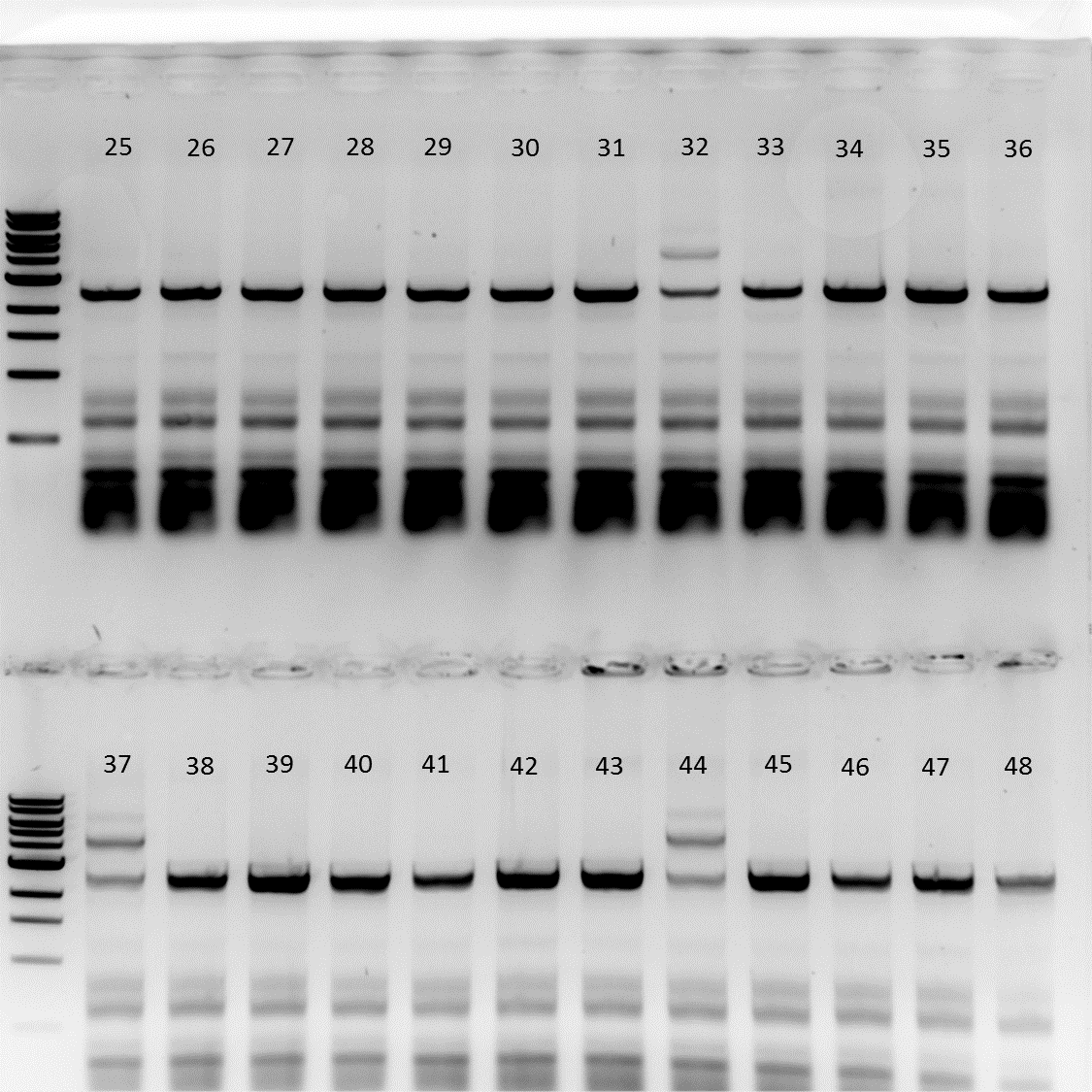

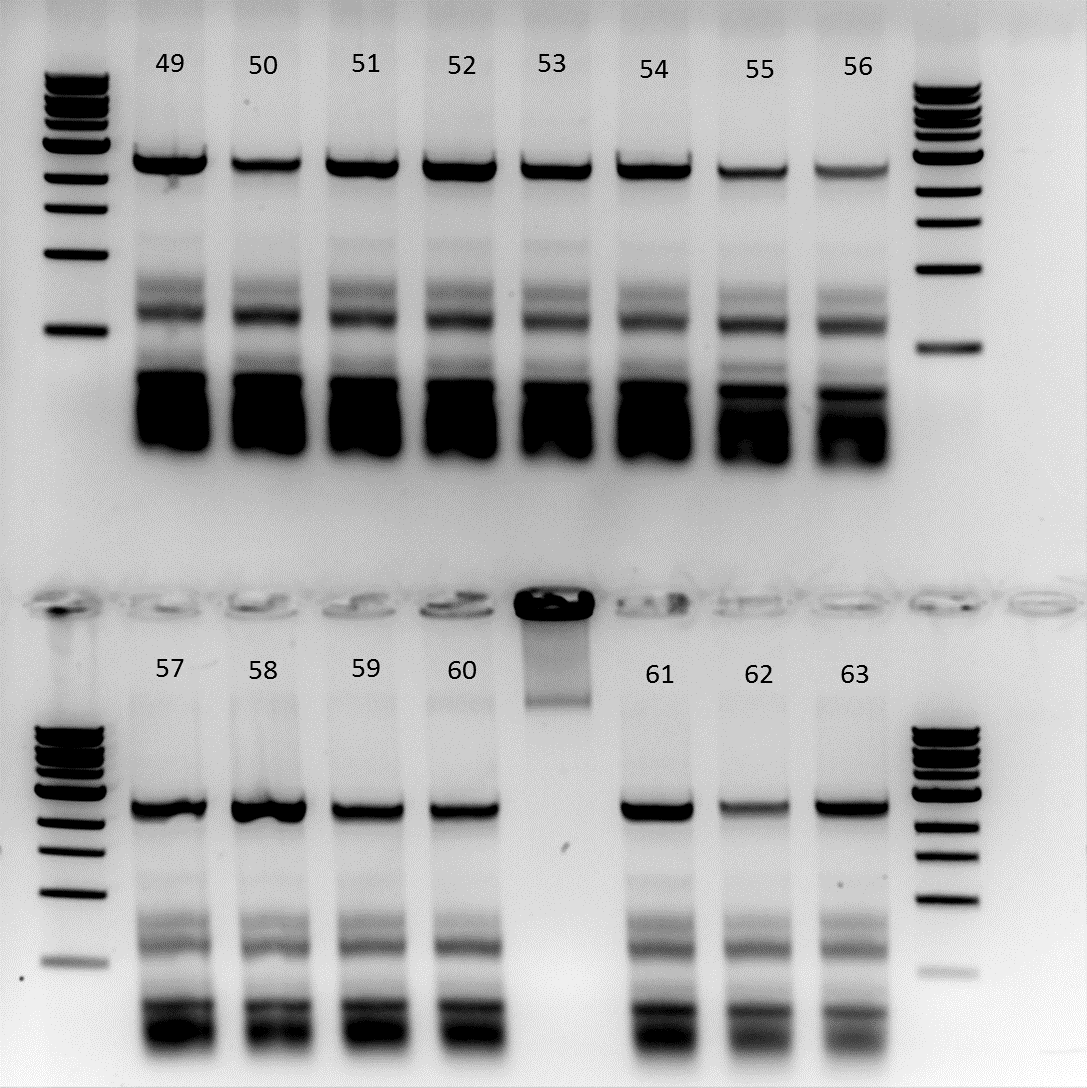


S2. cPCR results of 1kb HR transformants. A total of 63 zeocin resistant colonies were screened to check the presence of mutant colonies. The mutant colonies generates an amplicon of size 4337 bps while the wild type results in 2591 bps long fragment upon PCR. 57 colonies resulted in WT amplicon while 6 colonies indicated both mutant and WT bands.

**NEB 1kb ladder**

**NEB 1kb ladder**

**NEB 1kb ladder**

**NEB 1kb ladder**

**NEB 1kb ladder**

**NEB 1kb ladder**

**2kb**

**2kb**

**3kb**

**4kb**

**4kb**

**2kb**

**3kb**

**4kb**

**2kb**

**3kb**

**2kb**

**3kb**

**2kb**

**3kb**

**2kb**

**3kb**


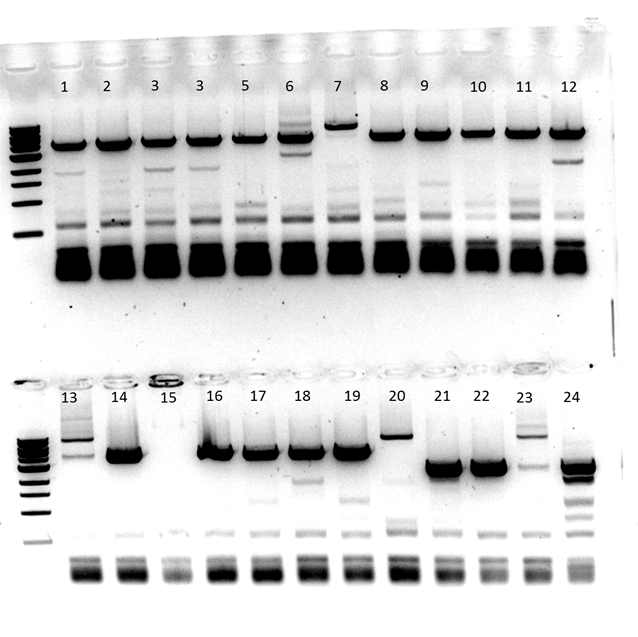

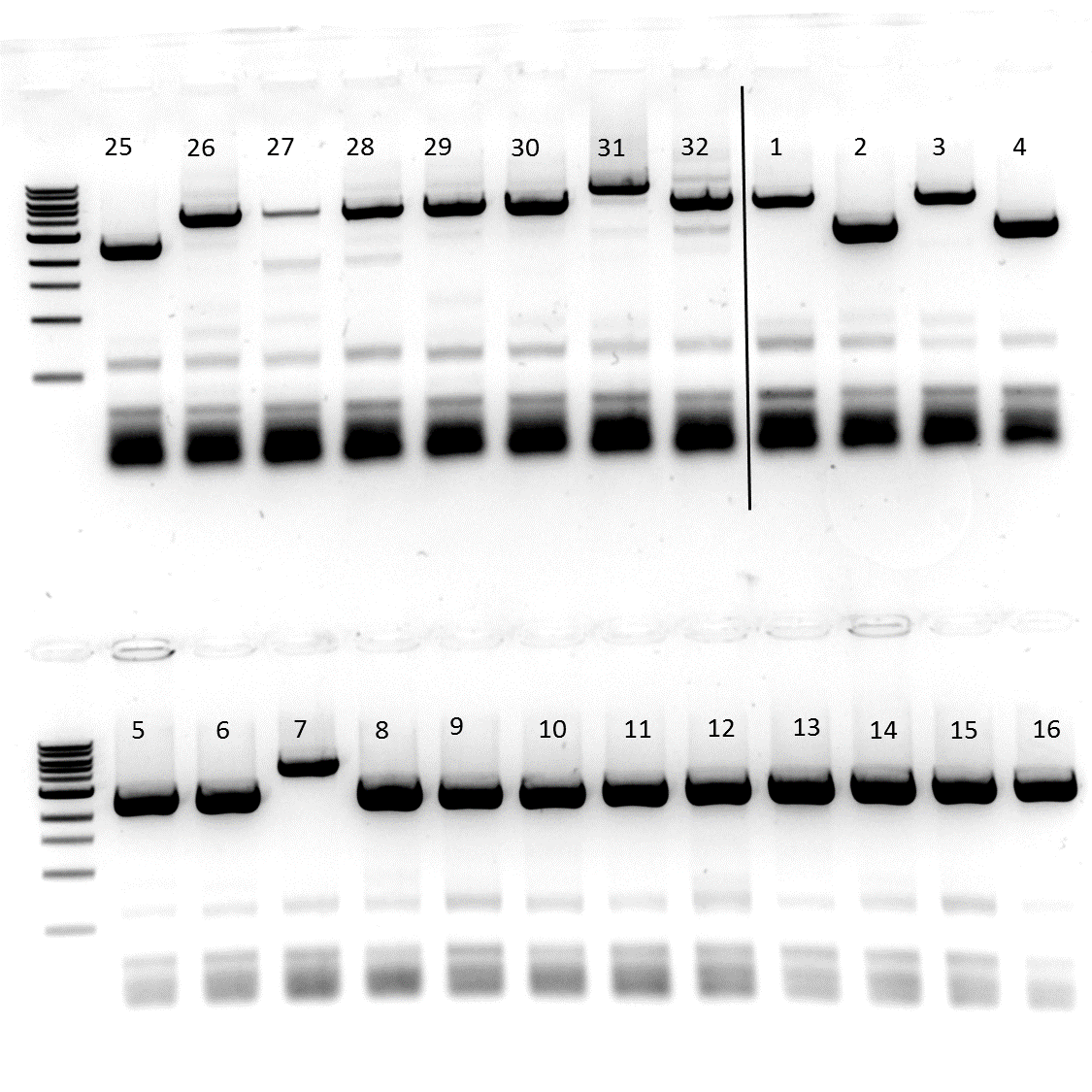


**NEB 1kb ladder**

**NEB 1kb ladder**

**NEB 1kb ladder**

**NEB 1kb ladder**

**4kb**

**4kb**

**4kb**

**4kb**

**2kb**

**3kb**

**2kb**

S4. cPCR results of FnCas12a-1 RNP based HDR. 30 colonies were screened and we observed that around 13 colonies produced the mutant band size of 4337bps.


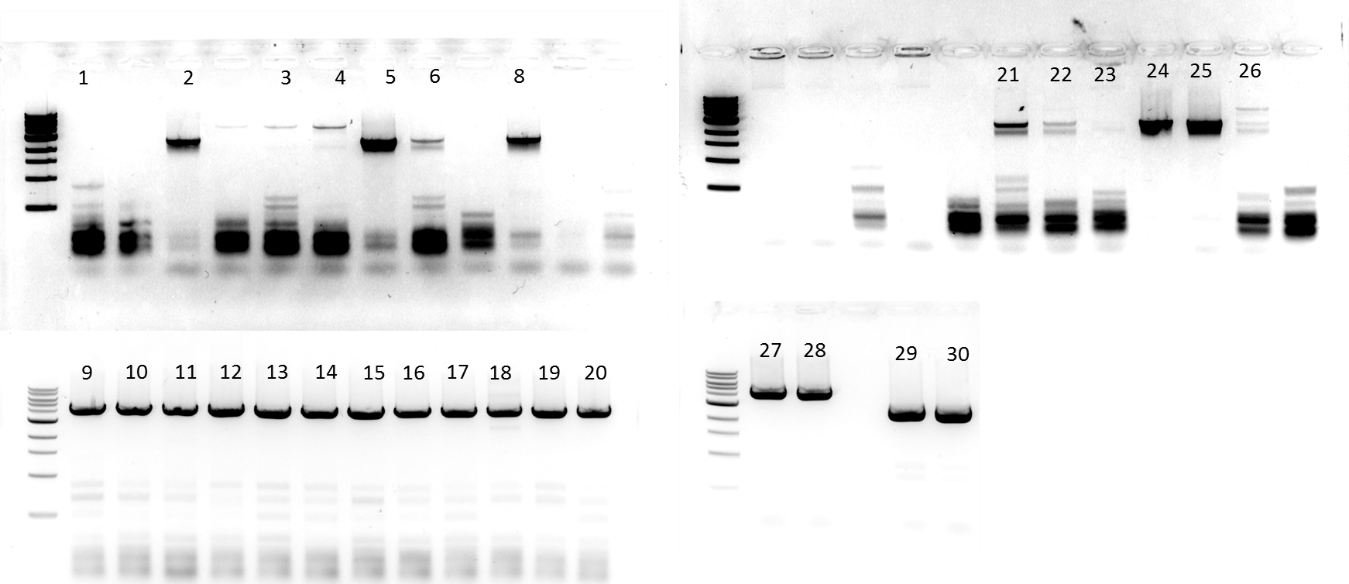


**NEB 1kb ladder**

**NEB 1kb ladder**

**NEB 1kb ladder**

**NEB 1kb ladder**

**3kb**

**4kb**

**2kb**

**3kb**

**2kb**

**3kb**

**2kb**

**3kb**


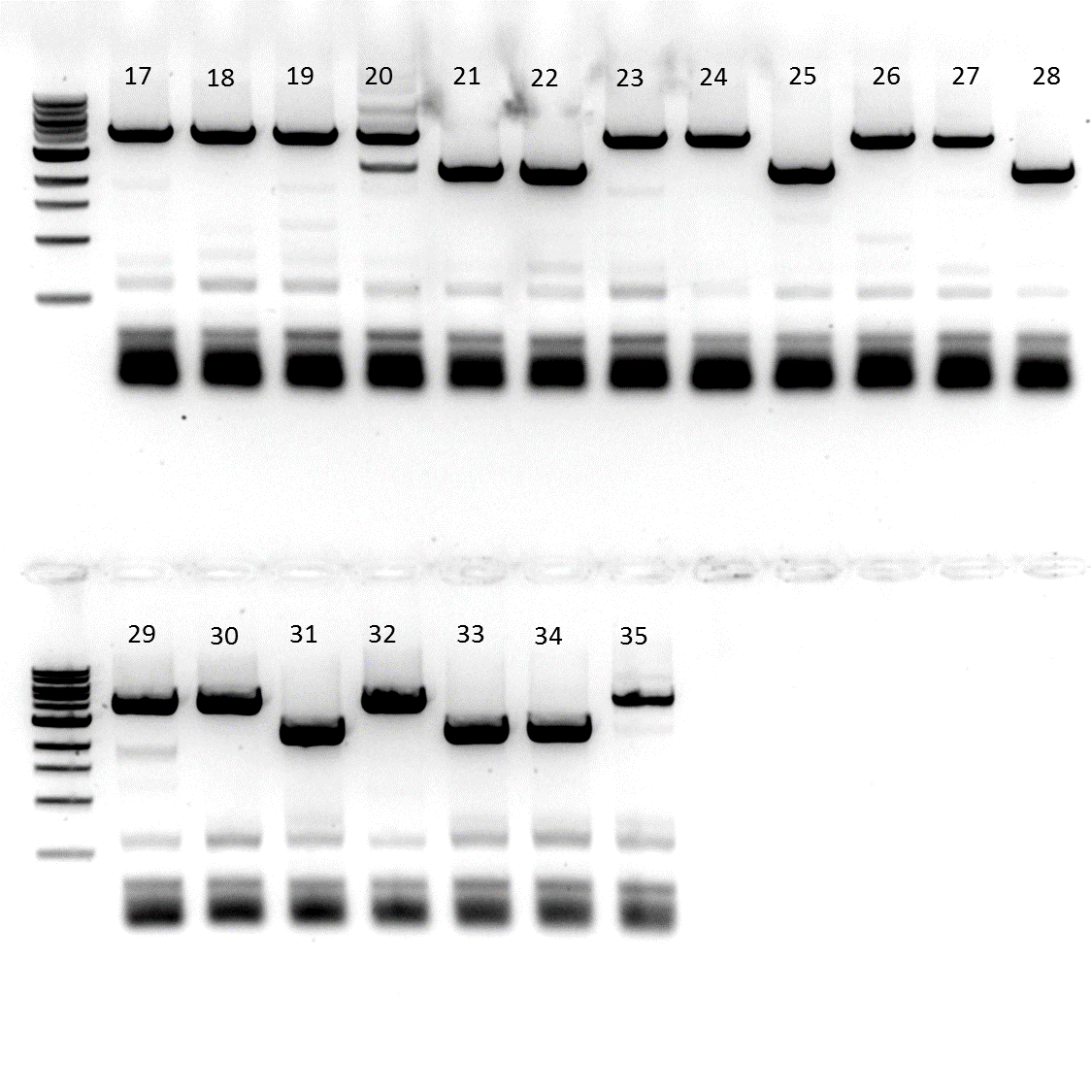


S3. cPCR results of Cas9 RNP based HDR. First 32 colonies are from transformants targeted with RNP harbouring guide 1 and next 35 colonies with guide 2. 22 out of 32 colonies screened for the NR targeting Cas RNP with guide 1 depicted mutant bands. For RNP with guide 2, among the 35 colonies screened 11 colonies were confirmed for mutant colonies.

**NEB 1kb ladder**

**NEB 1kb ladder**

**4kb**

**4kb**

**2kb**


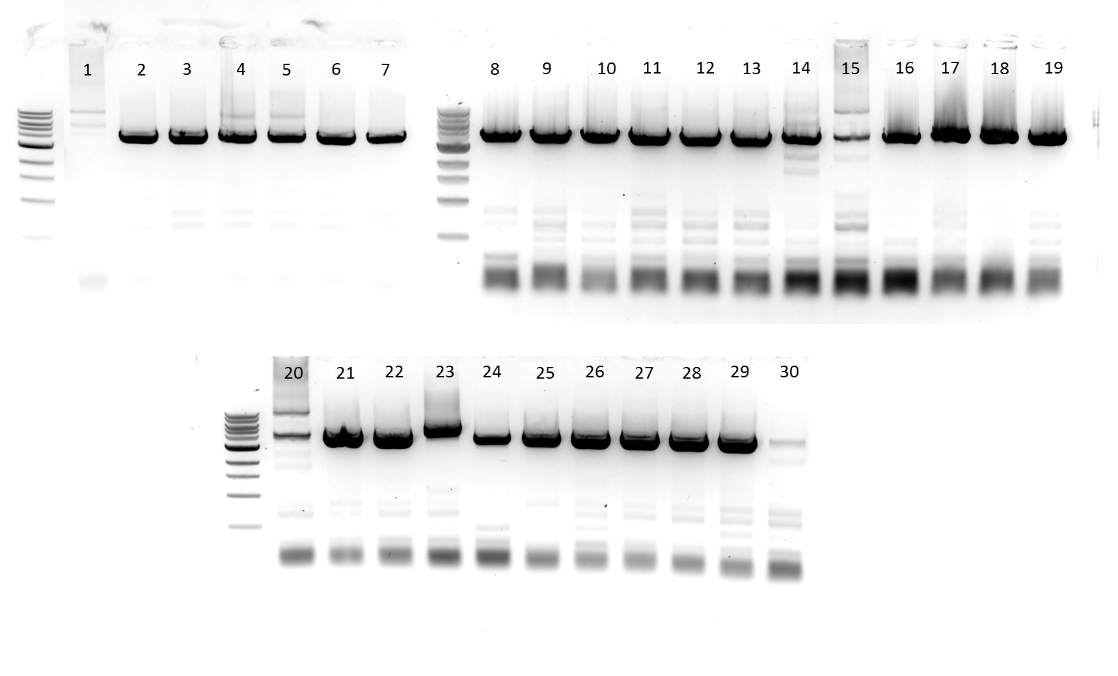


S5. cPCR results of FnCas12a-2 RNP based HDR. Out of the 30 colonies screened, 26 colonies were found to be the mutant colonies with the PCR band size of 4337 bps while the other 4 colonies indicated both the mutant and wild type (2591bps) bands

**NEB 1kb ladder**

**NEB 1kb ladder**

**NEB 1kb ladder**

**3kb**

**4kb**

**4kb**

**3kb**

**2kb**

**2kb**

**3kb**


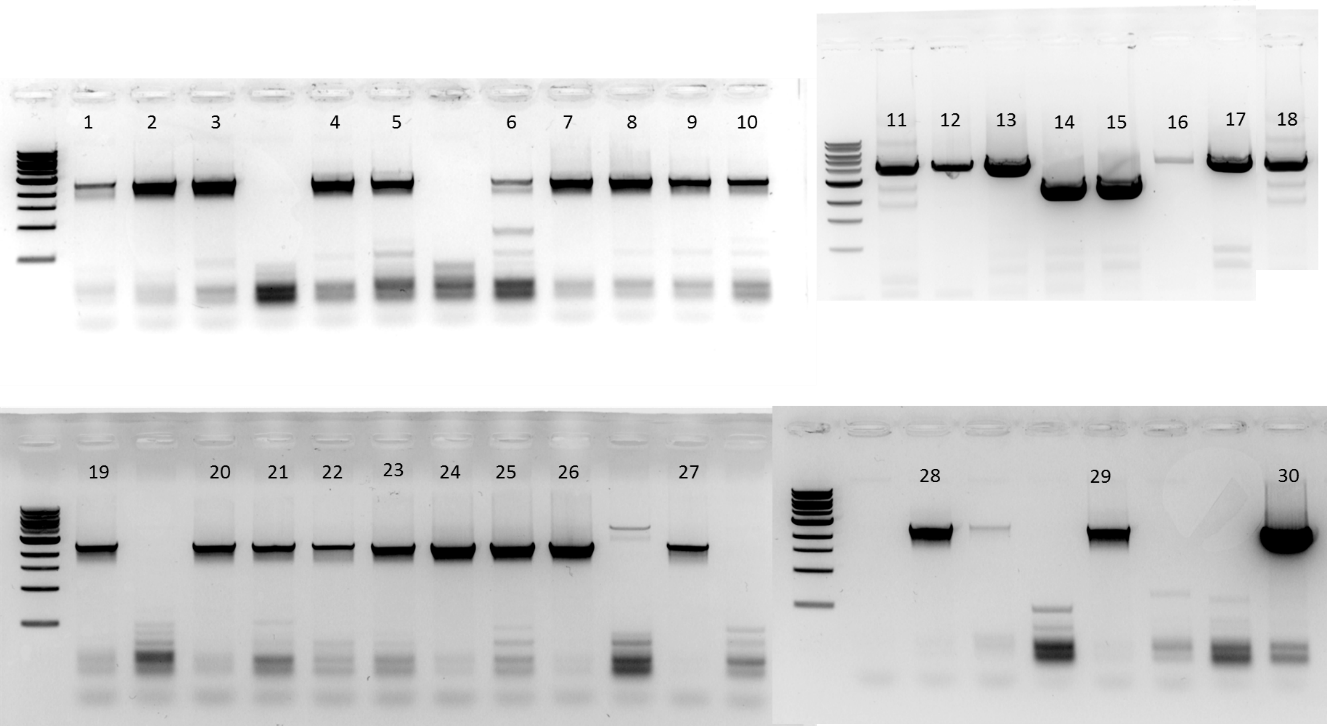


S6. cPCR results of FnCas12a-3 RNP based HDR. Only 6 colonies yielded mutant bands (4337bps) with this RNP among the 30 colonies screened

**2kb**

**3kb**

**4kb**

**4kb**


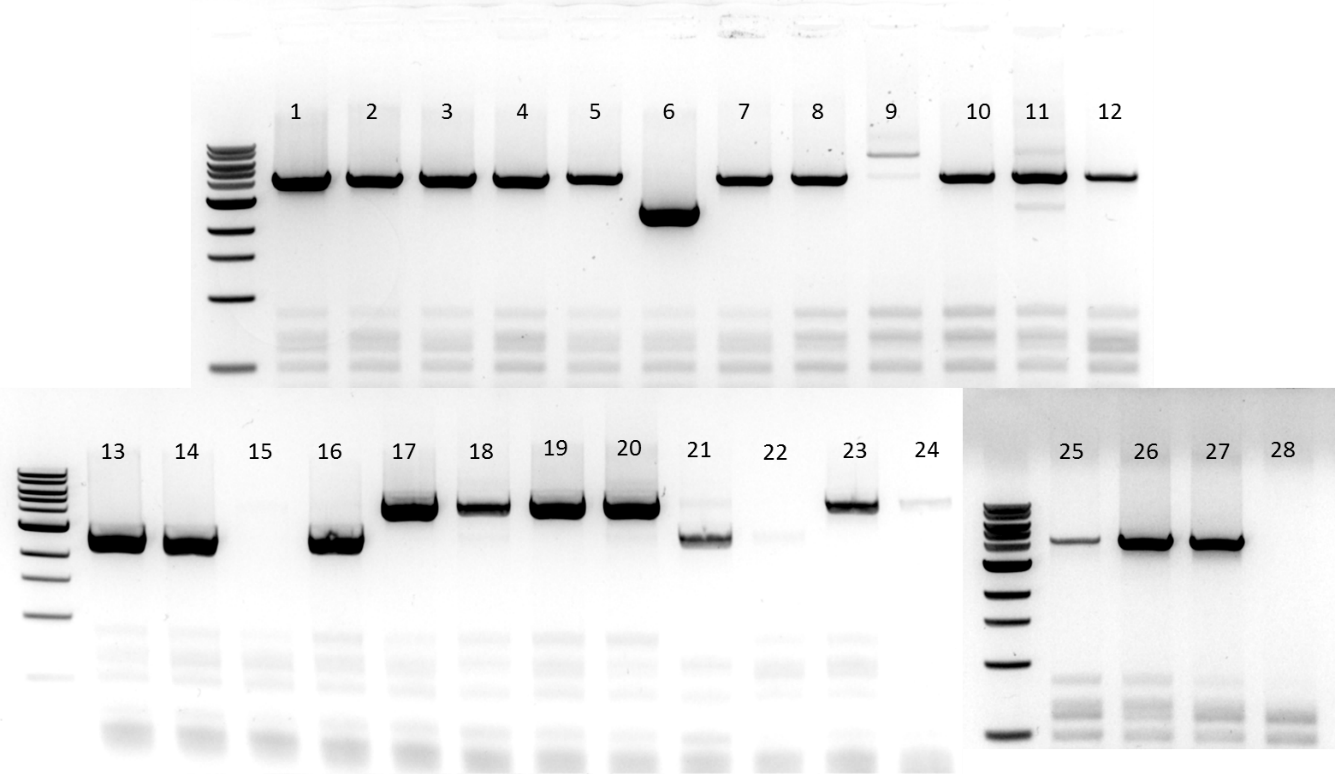


S7. cPCR results of LbCas12a-1 RNP based HDR. (cPCR 15,22 and 28 was repeated and we observed 22 was NR-KO while the other two were WT). 22 colonies among the 28 screened colonies indicated the mutant band size.

**NEB 1kb ladder**

**NEB 1kb ladder**

**NEB 1kb ladder**

**2kb**

**3kb**

**4kb**

**2kb**

**3kb**

**4kb**

S8. cPCR results of LbCas12a-2 RNP based HDR. 19 colonies out of 28 colonies indicated the mutant bands.


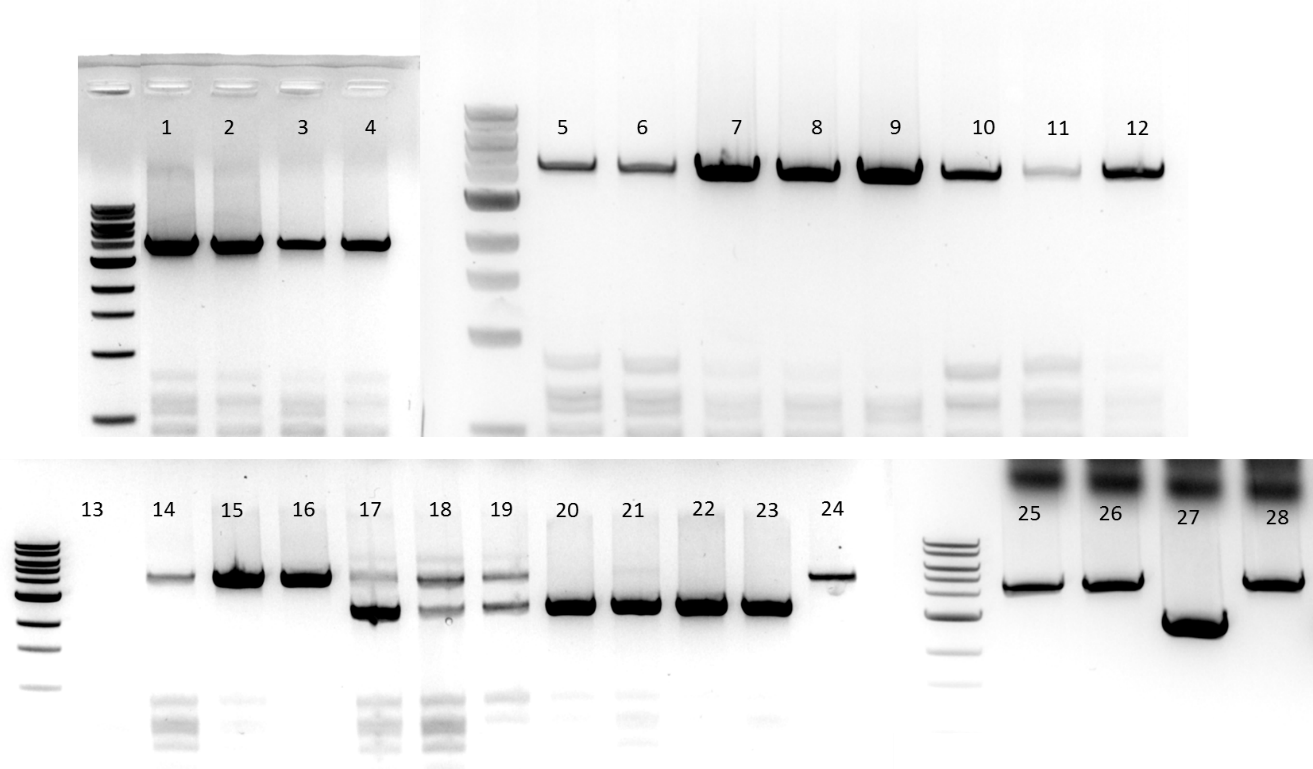


**NEB 1kb ladder**

**NEB 1kb ladder**

**NEB 1kb ladder**

**NEB 1kb ladder**

**2kb**

**3kb**

**4kb**

**2kb**

**3kb**

**4kb**


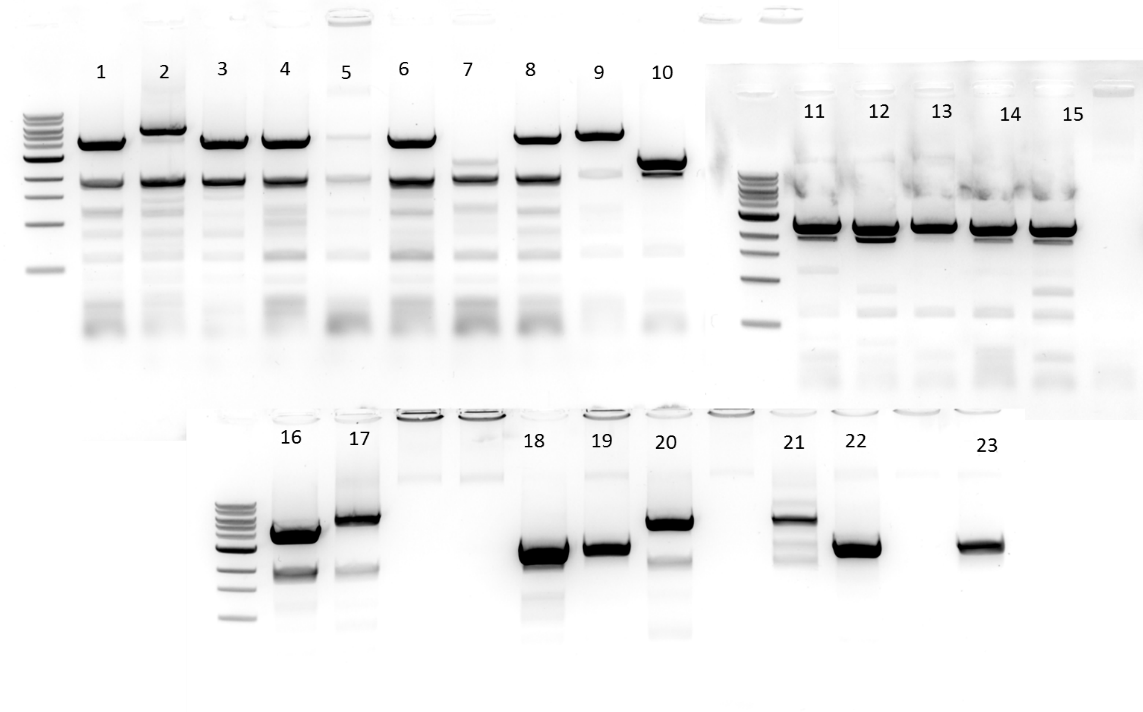


S9. cPCR results of LbCas12a-3 RNP based HDR. No mutant colonies were observed among the 23 colonies screened. However, 65% of the screened colonies indicated both the wild type and mutant bands.

**NEB 1kb ladder**

**NEB 1kb ladder**

**NEB 1kb ladder**

**2kb**

**3kb**

**4kb**

**2kb**

**3kb**

**4kb**


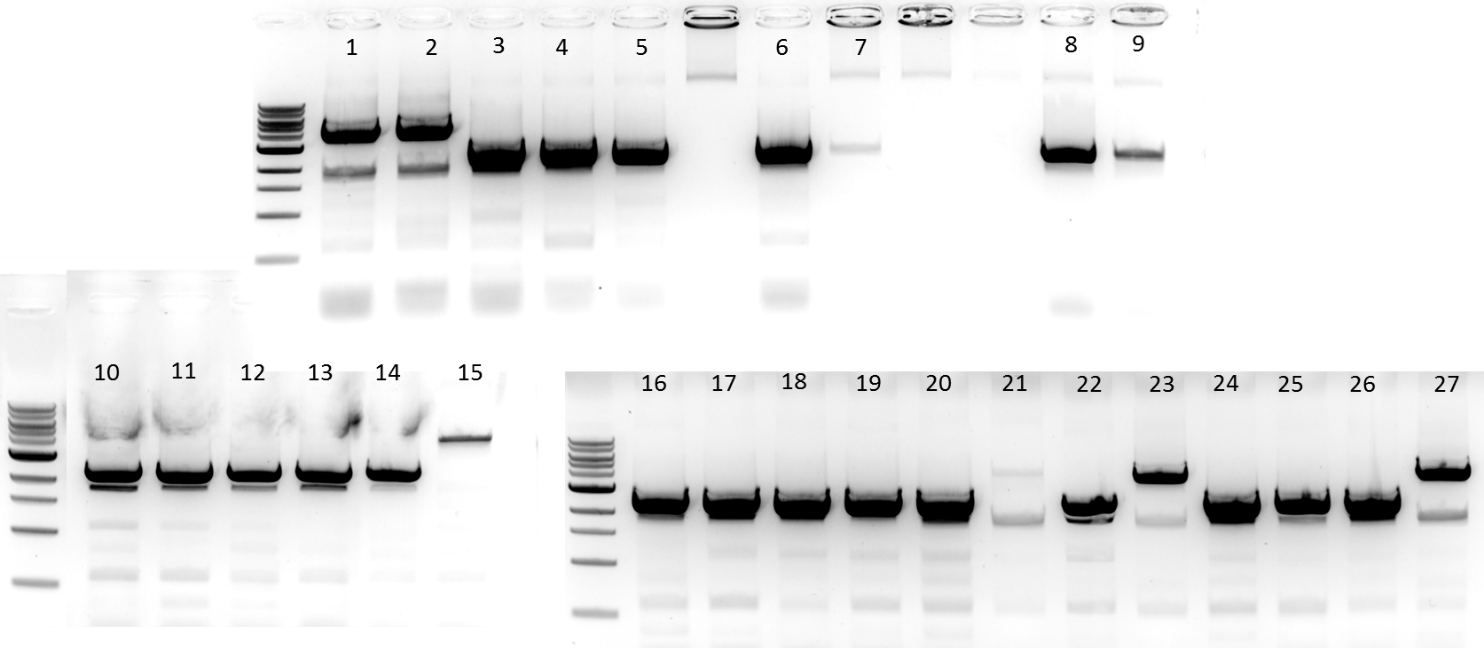


S10. cPCR results of AsCas12a-1 RNP based HDR. Obtained a single NR mutant colony among the 27 colonies screened

**NEB 1kb ladder**

**NEB 1kb ladder**

**NEB 1kb ladder**

**2kb**

**3kb**

**4kb**

**2kb**

**3kb**

**4kb**

S11. cPCR results of AsCas12a-2 RNP based HDR. No mutant colonies obtained among the 30 colonies screened.


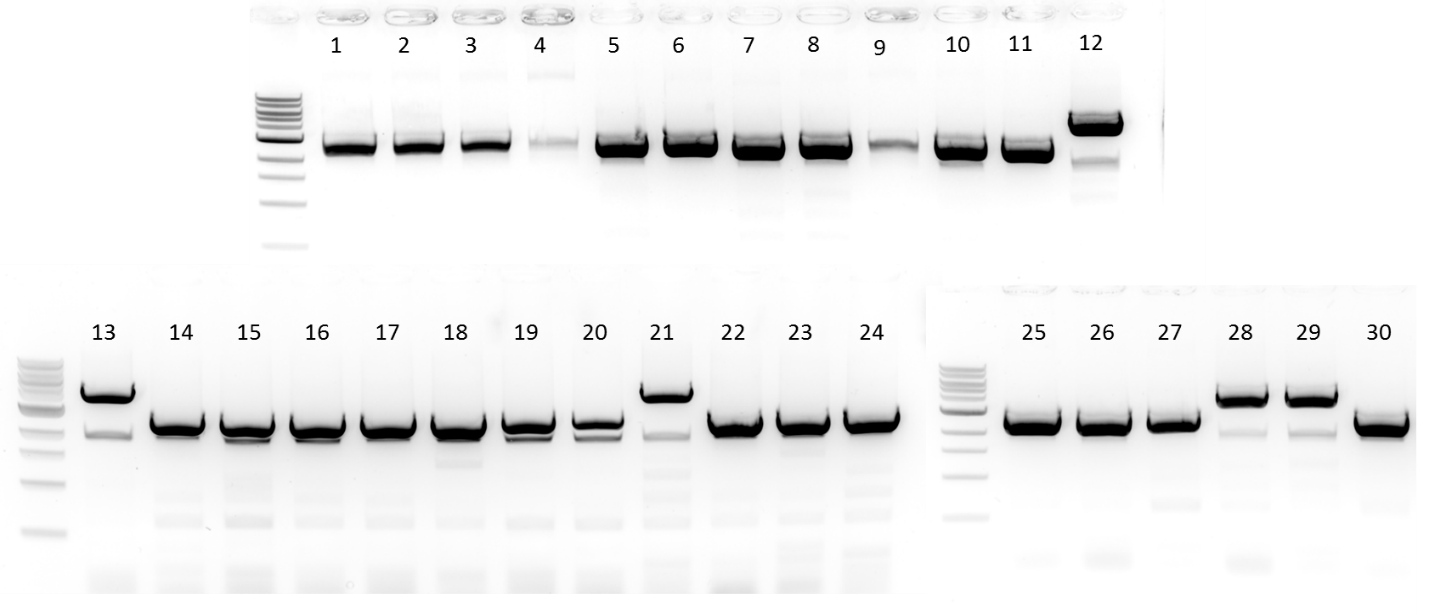


**NEB 1kb ladder**

**NEB 1kb ladder**

**NEB 1kb ladder**

**2kb**

**3kb**

**4kb**

**2kb**

**3kb**

**4kb**


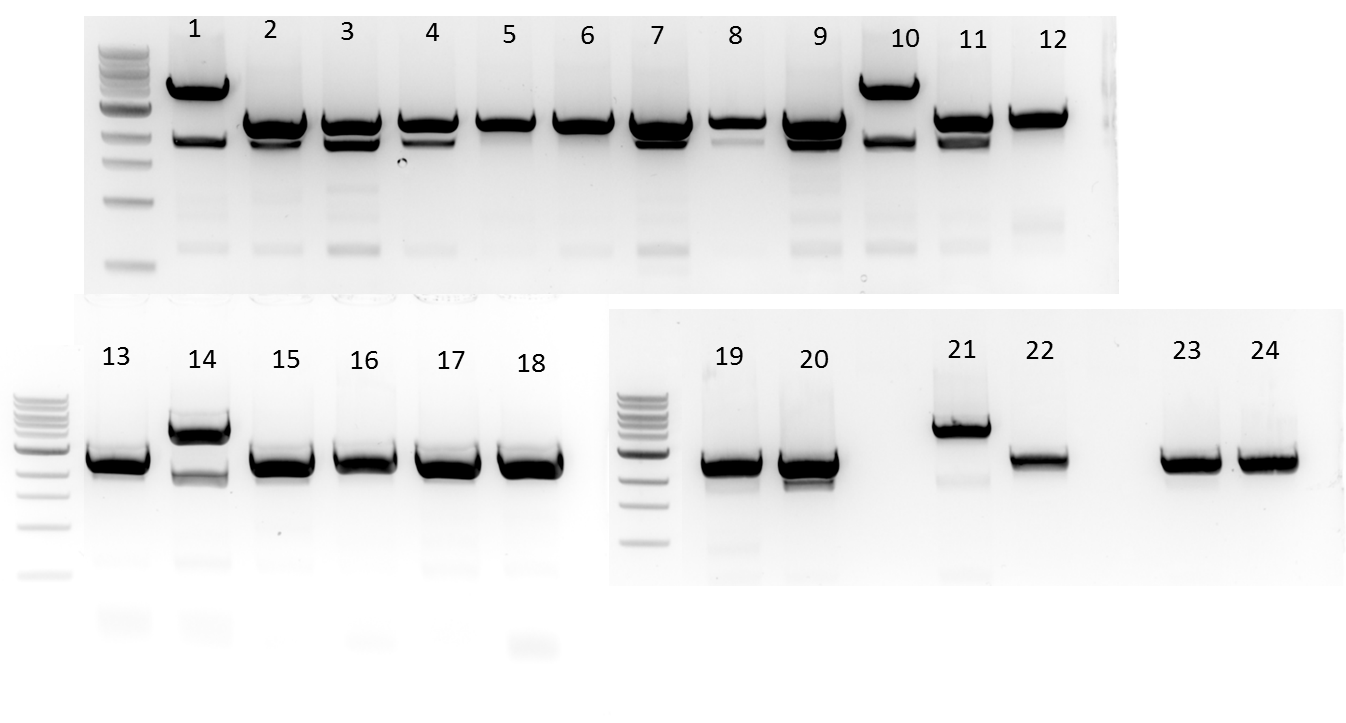


S12. cPCR results of AsCas12a-3 RNP based HDR. 1 mutant colony indicating the mutant band size of 4337bps was observed.

**NEB 1kb ladder**

**NEB 1kb ladder**

**NEB 1kb ladder**

**2kb**

**3kb**

**4kb**

**2kb**

**3kb**

**4kb**

S13. WT and NR-KO (circled) mutants re-streaked on to F/2 media with Ammonia (left) or nitrate (right) as the sole nitrogen source . The mutants were found to bleach on media with nitrate as the sole nitrogen source while they grew well on the plate with ammonia as the nitrogen source. The image was taken after 4 weeks upon re-streaking the colonies into fresh F/2 plates with appropriate N2 source.


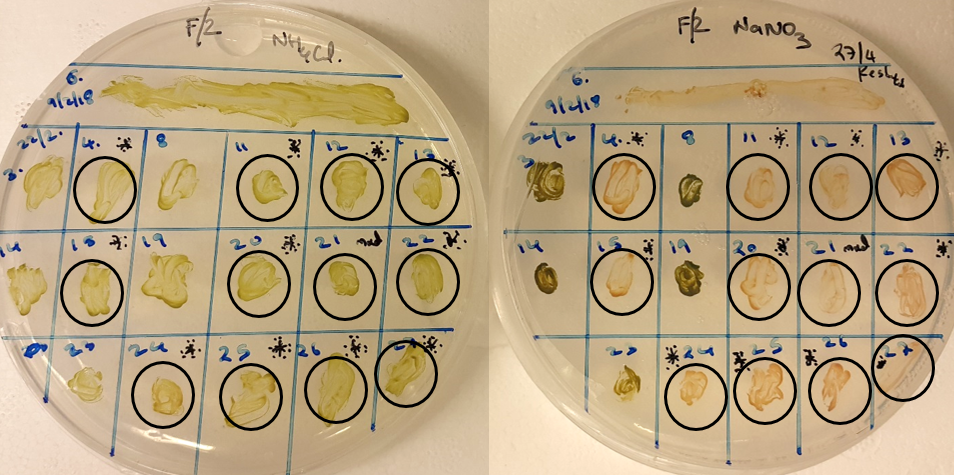

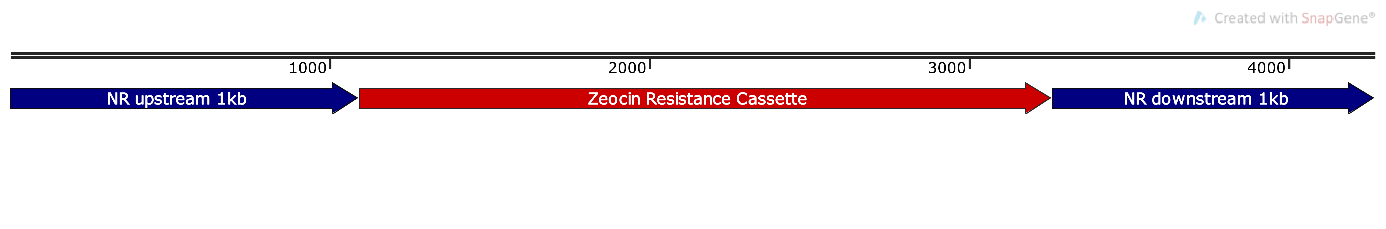


S14. Map of the linear DNA harbouring the HR flanks for NR gene deletion and zeocin resistance cassette.

S15. Map of the plasmid used for amplifying the linear DNA harbouring the HR flanks for NR gene deletion and zeocin resistance cassette. The linear DNA was amplified by PCR using primers that bind at the 5’ end of upstream HR flank and 3’ end of the downstream HR flank. Genbank file of the above plasmid is provided below.


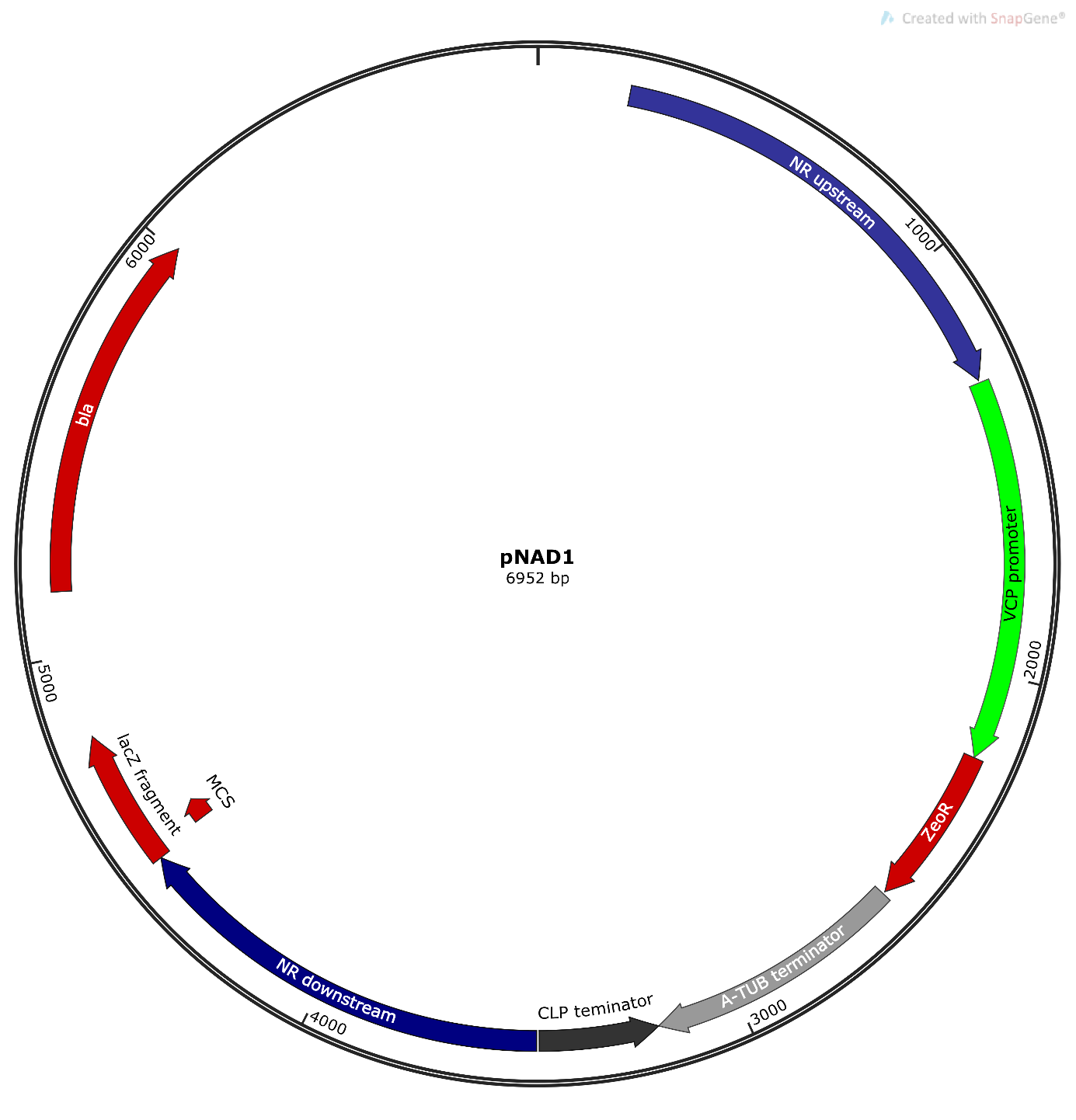


LOCUS pNAD1 6952 bp DNA circular SYN 11-JAN-2019

DEFINITION pUC cloning vector containing the upstream and downstream HR flanks

of NR gene with zeocin resistance cassette

ACCESSION pNAD1

KEYWORDS .

SOURCE Unknown.

ORGANISM Unknown

Unclassified.

REFERENCE 1 (bases 1 to 6952)

AUTHORS Self

JOURNAL Unpublished.

COMMENT SECID/File created by Clone Manager, Scientific & Educational Software

FEATURES Location/Qualifiers

misc_feature 216..1302

/gene="NR upstream "

promoter 1309..2200

/gene="VCP promoter"

CDS 2201..2575

/gene="ZeoR"

/product="confers resistance to antibiotic zeocin"

terminator 2582..3192

/gene="A-TUB terminator"

terminator complement(3193..3472)

/gene="CLP teminator"

misc_feature 3479..4481

/gene="NR downstream"

CDS 4482..4805

/codon_start=1

/gene="lacZ fragment"

/product="LacZ-alpha fragment of beta-galactosidase"

/label=lacZ-alpha

/translation="MTMITPSLHACRSTLEDPRVPSSNSLAVVLQRRDWENPGVTQLN

RLAAHPPFASWRNSEEARTDRPSQQLRSLNGEWRLMRYFLLTHLCGISHRIWCTLSTI

CSDAA"

misc_feature 4499..4555

/label=MCS

/note="pUC18/19 multiple cloning site"

CDS 5151..6011

/codon_start=1

/gene="bla"

/product="beta-lactamase"

/label=AmpR

/note="confers resistance to ampicillin, carbenicillin,

and related antibiotics"

/translation="MSIQHFRVALIPFFAAFCLPVFAHPETLVKVKDAEDQLGARVGY

IELDLNSGKILESFRPEERFPMMSTFKVLLCGAVLSRIDAGQEQLGRRIHYSQNDLVE

YSPVTEKHLTDGMTVRELCSAAITMSDNTAANLLLTTIGGPKELTAFLHNMGDHVTRL

DRWEPELNEAIPNDERDTTMPVAMATTLRKLLTGELLTLASRQQLIDWMEADKVAGPL

LRSALPAGWFIADKSGAGERGSRGIIAALGPDGKPSRIVVIYTTGSQATMDERNRQIA

EIGASLIKHW"

ORIGIN

1 gcgcccaata cgcaaaccgc ctctccccgc gcgttggccg attcattaat gcagctggca

61 cgacaggttt cccgactgga aagcgggcag tgagcgcaac gcaattaatg tgagttagct

121 cactcattag gcaccccagg ctttacactt tatgcttccg gctcgtatgt tgtgtggaat

181 tgtgagcgga taacaatttc acacaggaaa cagctctggc gatcgttgag actaaccgcg

241 gttgtaagcc ggaaccagct gttgttcatc actgtgggta gggaagggag gggtggaaag

301 gggagttcag gggtatgaat aaggtgtgac gattactctt actctcatag acacaggtaa

361 agatagagat ggatggaata aaagtatctc tagaagagag gaaacgaaat actcactgcc

421 taggacgttc cacgtcaagt ctcgaggctg tgtgttctgc gagttgtccc atgcacgaca

481 ggcgacattc gtacagccca cctatgagag agggagggat tgtatatatg agagcgtgat

541 ggaacacaga cgtttctcgc tcgttctcaa gctgcactca ccagacgtag gatgtcgaca

601 tcaagattcc aaatacgcca gcaccagaag cggccgtgtt cagttgggta ctcctcactg

661 ctcagctgag ctagttccca gctctactcg tggtgcgtag tcggaatggg tgtgtaaatg

721 tcgattgagt caatgatagc agtgtgaaaa ccgcgacaag gtgatacttc ctaccttgcc

781 atcgtcgagg gagacctcca tgcgttgcac ttgccggccg ccaccacagt aggcgtagcc

841 ttggagtttg taagtgcctt tcttcagcgg gatctcctcg tcgtgagtct ggcagggaaa

901 atgacgaaca gcagtataag cggcgagctt gcacgtttgt aaactttcgt aatcccagct

961 gaggcccgcg gacacgagta cgtaccggat gggtgatggc cgagttaagg ttgaggtcgt

1021 tgatgatgta ctcaggcttg cgccaccagc cctcattatc cgcgcgctct tggtccaccg

1081 acgagggcag gacgcgattg tcgtggaagt ggtaaaagga ctgcgactct tggcggctga

1141 tagtgatgcg agtaagccat ttgatcatcc gtccccctat gtaccctgca agtagaggag

1201 ataaaaagga aagtgagtaa tatggctgtg gttcatgtat gtatgtgcgt gtatgggtgt

1261 gtatgtgtat gtttgattgt gtacctggaa taatgaggcg gagttggagg ggtcgtttat

1321 ttctctgcca gccttggacc tcgtggttgg gcgtttcctc ctccttggtg cactgaacct

1381 gtccgcatcc tgcgtcggtt tggcggtctt ttgtcctttc ctctatagcc cgcccgtcta

1441 gagggcacac gcgatgatct ttatatctct tcatgtgtct ttgttttaac taggatactg

1501 ccgggtgaat gcccatcaga caagaggcca aactctatct acaccctttt gacttctgtt

1561 gtggtcgtag tgtgtgcttg catgccctga aagtccaggc atcccacttg tgctctaacc

1621 ccattcaaaa cagcagaagt gcttaattaa gatatagatt catgatctcc tgtcccctcc

1681 ttcttacctt ttcacaaacc tcacacagaa gtctccactc ttcgcctcta aaacctcttt

1741 ttaaattatg gtaagttcgt gcggcagtgg gttttcggat ctatatttgt caagatccag

1801 ttcaaggtca gggatgtaga ttaagtacag aaggagaagc acaagcgcgc cagttcgccc

1861 ctcacggcct ggagcagggc atttaatccc tctatcttac cagaaccata ctatacaacc

1921 aatcctgttg gcatcgctct gtctatttgt cgtgcgtgca tgtgtccatg gtgtggtggg

1981 gggcaggggt tttcggggtt gcggttgaag gcaccttatc agaaagatgc cctcagagat

2041 agaggtagcc ccctcccccc gatcttcgac cagtcctgtc aggcgaacac tttcacccgt

2101 cgttcacctc gttacacaca aggagtagac ctctgaagtt ctaattgtca taaatgcccc

2161 tcccccctcc ctccttccct tcatcctccc ctccgagcag atggccaagt tgaccagtgc

2221 cgttccggtg ctcaccgcgc gcgacgtcgc cggagcggtc gagttctgga ccgaccggct

2281 cgggttctcc cgggacttcg tggaggacga cttcgccggt gtggtccggg acgacgtgac

2341 cctgttcatc agcgcggtcc aggaccaggt ggtgccggac aacaccctgg cctgggtgtg

2401 ggtgcgcggc ctggacgagc tgtacgccga gtggtcggag gtcgtgtcca cgaacttccg

2461 ggacgcctcc gggccggcca tgaccgagat cggcgagcag ccgtgggggc gggagttcgc

2521 cctgcgcgac ccggccggca actgcgtgca cttcgtggcc gaggagcagg actgatggcc

2581 agggatcagg aggagggagt gaagaggaga agggatctgg tttcagagat ccccacttct

2641 gccgtcgtct ttcggccttc cttcctttta ggtgtcatgc cttaggtcct tcaagtcctc

2701 acctgtcgtc gtcatgtgtg tgtgtgcccg tcatacaagt cactcgatcc aattcacgca

2761 tcggttcaat caaaataaga ctagaccccg agggaagaag ggcagaagga aatcgaaggg

2821 gtgggatgtg tgtgagagag ggaaggagaa atgaaagaag tgaacaatgt catggtagcc

2881 agtaaggaga gagtagaagc gaagaaagca aaagcactgt tgtgaagaaa cgaaatggaa

2941 gatggtcatc gctcctggct ctacttgtgg tttttctatc tttaatttca ggcgtcctgg

3001 tctcgttaca tcagctccct tatctcattg gtttatcccc tactctactg ctgcttcttc

3061 cttccatccg tgactgtata acaacgaatt gtagtaccgc agatagacaa tagaaaaatg

3121 ccaaaaaagg catcattgat ttgctcctcc ccattaagtc actgtacgcc accatcgcca

3181 ctaccctccc ttagcaaata gtctttattt agaacacaga agaagagtac gttgtgtgca

3241 tgcgtgttgt tcgtacaact tccgtgtcag gacgagagac catttcaaca gtgagtaatt

3301 ccgcctgcgt ttattttttc ctctttttcc ttttcttgtt ttcttccttt gtacgtgtat

3361 ttccctctct tccacgtctt ctttatcgtc atcttgtctg ccatccttac tctatcttct

3421 accctcactc acttccacac tctcaagtcc accctccccc tccccctcac tctccaacgg

3481 cgaccagttc gtccattgac aaaacctggg gctcagctac gccaaggccg gtcacggtta

3541 tgcggtggtc atcccaggca agcttgggta ctgggccgtg gttgctgtag gtgatggtgt

3601 agtttagaaa gatgatgaga gacacgaact ggattggccg acccagaaaa gaaggactta

3661 ccgcacatag tgcatggcgg cgggggtaat gaagccagcg tcgaccagct ccttggtgtg

3721 cggctcggcg ttaaacgggt ggcgacccgt caggcggatc atgcctggca ggcgctgaac

3781 ccacttgtct gccgtgtcat cgtctcgcgc gtctgtactg gtggcacgga ccggctcagg

3841 cgccgtaggc acctgtggtg agagtttaaa ggccatgtag aggagcaacg tgctaaagct

3901 gctgcggcac ctgccctcgt tgtgtggtgg gtgggtcatg aagtgtggtc agagtcgggt

3961 cagtcatagc tgtccagagt cgacgtatgc agaaacgaga acacatattg tcgattcctg

4021 ctgctacgac tgcgcgctaa gatccggcgc cttgcagcgg ttgcttttcc tgttccgaca

4081 cgtcatgttc gcggccccca atcgaggcgg cggttttttg gtgcagtgcc tgttgtgcca

4141 acgccgtcgg tgataaagta acaggtaggt ttttggcaag gagttacaca aaccagtcga

4201 ccaggggaca tgaaatagaa ctttaccttt cgcaatcgtg gatacgtctt gtggggtacg

4261 gccgagaagt ggcgcaacaa aaaaagtatg tttccatggt gacccgcgaa ccggggcagg

4321 gatgtcgaca taaattgaaa acccgccgat tttcaaccgg acttagccaa cgtggtcatc

4381 tccagttact cttgagtggg ggtattatga tttgtcttgc ttgtttgctt ctttatgaaa

4441 gcttggcatt gttttttcca aagcacctaa tgacatgccg catgaccatg attacgccaa

4501 gcttgcatgc ctgcaggtcg actctagagg atccccgggt accgagctcg aattcactgg

4561 ccgtcgtttt acaacgtcgt gactgggaaa accctggcgt tacccaactt aatcgccttg

4621 cagcacatcc ccctttcgcc agctggcgta atagcgaaga ggcccgcacc gatcgccctt

4681 cccaacagtt gcgcagcctg aatggcgaat ggcgcctgat gcggtatttt ctccttacgc

4741 atctgtgcgg tatttcacac cgcatatggt gcactctcag tacaatctgc tctgatgccg

4801 catagttaag ccagccccga cacccgccaa cacccgctga cgcgccctga cgggcttgtc

4861 tgctcccggc atccgcttac agacaagctg tgaccgtctc cgggagctgc atgtgtcaga

4921 ggttttcacc gtcatcaccg aaacgcgcga gacgaaaggg cctcgtgata cgcctatttt

4981 tataggttaa tgtcatgata ataatggttt cttagacgtc aggtggcact tttcggggaa

5041 atgtgcgcgg aacccctatt tgtttatttt tctaaataca ttcaaatatg tatccgctca

5101 tgagacaata accctgataa atgcttcaat aatattgaaa aaggaagagt atgagtattc

5161 aacatttccg tgtcgccctt attccctttt ttgcggcatt ttgccttcct gtttttgctc

5221 acccagaaac gctggtgaaa gtaaaagatg ctgaagatca gttgggtgca cgagtgggtt

5281 acatcgaact ggatctcaac agcggtaaga tccttgagag ttttcgcccc gaagaacgtt

5341 ttccaatgat gagcactttt aaagttctgc tatgtggcgc ggtattatcc cgtattgacg

5401 ccgggcaaga gcaactcggt cgccgcatac actattctca gaatgacttg gttgagtact

5461 caccagtcac agaaaagcat cttacggatg gcatgacagt aagagaatta tgcagtgctg

5521 ccataaccat gagtgataac actgcggcca acttacttct gacaacgatc ggaggaccga

5581 aggagctaac cgcttttttg cacaacatgg gggatcatgt aactcgcctt gatcgttggg

5641 aaccggagct gaatgaagcc ataccaaacg acgagcgtga caccacgatg cctgtagcaa

5701 tggcaacaac gttgcgcaaa ctattaactg gcgaactact tactctagct tcccggcaac

5761 aattaataga ctggatggag gcggataaag ttgcaggacc acttctgcgc tcggcccttc

5821 cggctggctg gtttattgct gataaatctg gagccggtga gcgtgggtct cgcggtatca

5881 ttgcagcact ggggccagat ggtaagccct cccgtatcgt agttatctac acgacgggga

5941 gtcaggcaac tatggatgaa cgaaatagac agatcgctga gataggtgcc tcactgatta

6001 agcattggta actgtcagac caagtttact catatatact ttagattgat ttaaaacttc

6061 atttttaatt taaaaggatc taggtgaaga tcctttttga taatctcatg accaaaatcc

6121 cttaacgtga gttttcgttc cactgagcgt cagaccccgt agaaaagatc aaaggatctt

6181 cttgagatcc tttttttctg cgcgtaatct gctgcttgca aacaaaaaaa ccaccgctac

6241 cagcggtggt ttgtttgccg gatcaagagc taccaactct ttttccgaag gtaactggct

6301 tcagcagagc gcagatacca aatactgttc ttctagtgta gccgtagtta ggccaccact

6361 tcaagaactc tgtagcaccg cctacatacc tcgctctgct aatcctgtta ccagtggctg

6421 ctgccagtgg cgataagtcg tgtcttaccg ggttggactc aagacgatag ttaccggata

6481 aggcgcagcg gtcgggctga acggggggtt cgtgcacaca gcccagcttg gagcgaacga

6541 cctacaccga actgagatac ctacagcgtg agctatgaga aagcgccacg cttcccgaag

6601 ggagaaaggc ggacaggtat ccggtaagcg gcagggtcgg aacaggagag cgcacgaggg

6661 agcttccagg gggaaacgcc tggtatcttt atagtcctgt cgggtttcgc cacctctgac

6721 ttgagcgtcg atttttgtga tgctcgtcag gggggcggag cctatggaaa aacgccagca

6781 acgcggcctt tttacggttc ctggcctttt gctggccttt tgctcacatg ttctttcctg

6841 cgttatcccc tgattctgtg gataaccgta ttaccgcctt tgagtgagct gataccgctc

6901 gccgcagccg aacgaccgag cgcagcgagt cagtgagcga ggaagcggaa ga

//
